# Hydrogen sulfide alleviates uremic cardiomyopathy by regulating PI3K/PKB/mTOR-mediated overactive autophagy in 5/6 nephrectomy mice

**DOI:** 10.3389/fphar.2022.1027597

**Published:** 2022-12-15

**Authors:** Jianan Feng, Han Li, Shixiang Wang

**Affiliations:** Department of Nephrology, Beijing Chao-Yang Hospital, Capital Medical University, Beijing, China

**Keywords:** uremic cardiomyopathy, hydrogen sulfide, cardiomyocytes, autophagy, PI3K/PKB /mTOR signal

## Abstract

The gasotransmitter hydrogen sulfide (H_2_S) plays important physiological and pathological roles in the cardiovascular system. However, the involvement of H_2_S in recovery from uremic cardiomyopathy (UCM) remains unclear. This study aimed to determine the therapeutic efficacy and elucidate the underlying mechanisms of H_2_S in UCM. A UCM model was established by 5/6 nephrectomy in 10-week-old C57BL/6 mice. Mice were treated with sodium hydrosulfide (NaHS, H_2_S donor), L-cysteine [L-Cys, cystathionine gamma-lyase (CSE) substrate], and propargylglycine (PPG, CSE inhibitor). Treatment of H9C2 cardiomyocytes utilized different concentrations of uremic serum, NaHS, PPG, and PI3K inhibitors (LY294002). Mouse heart function was assessed by echocardiography. Pathological changes in mouse myocardial tissue were identified using hematoxylin and eosin and Masson’s trichrome staining. Cell viability was assessed using the Cell Counting Kit-8. The protein expressions of CSE, p-PI3K, PI3K, p-PKB, PKB, p-mTOR, mTOR, and autophagy-related markers (Beclin-1, P62, and LC3) were detected using Western blotting. We found that NaHS and L-Cys treatment attenuated myocardial disarray, fibrosis, and left ventricular dysfunction in UCM mice. These abnormalities were further aggravated by PPG supplementation. Enhanced autophagy and decreased phosphorylation of PI3K, PKB, and mTOR protein expression by UCM were altered by NaHS and L-Cys treatment. *In vitro*, uremic serum increased overactive autophagy and decreased the phosphorylation levels of PI3K, PKB, and mTOR in cardiomyocytes, which was substantially exacerbated by endogenous H_2_S deficiency and attenuated by pre-treatment with 100 µm NaHS. However, the protective effects of NaHS were completely inhibited by LY294002. These findings support a protective effect of H_2_S exerted against UCM by reducing overactive autophagy through activation of the PI3K/PKB/mTOR pathway.

## 1 Introduction

Uremic cardiomyopathy (UCM) is a common complication in patients with chronic kidney disease (CKD), especially dialysis patients, which is associated with high mortality (Amador-Martínez et al., 2021). The characteristic pathological changes of UCM are left ventricular hypertrophy (LVH) and diastolic dysfunction in heart failure with preserved ejection fraction (HFpEF) phase. Subsequently, ventricular interstitial fibrosis, capillary thinning, and systolic dysfunction occur in heart failure with reduced ejection fraction (HFrEF) phase (Husain-Syed et al., 2015; Powers et al., 2016; Wang et al., 2017; Sárközy et al., 2018). The pathogenesis of UCM is multifactorial and complicated, involving hypertension, volume overload, activation of the renin-angiotensin-aldosterone system, stimulation of the β-adrenergic system, and insulin resistance. In addition, some specific factors that arise in uremia also play an important role, such as hyperparathyroidism, hyperphosphatemia, 1,25-dihydroxy-vitamin D deficiency, increased production of fibroblast growth factor 23, Klotho deficiency, circulating uremic toxins, and increases in endogenous cardiotonic steroid concentration (Vanholder et al., 2014; Spoto et al., 2016; Hung et al., 2017; Wang and Shapiro, 2019; Memmos and Papagianni, 2021).

Autophagy is a process that devours cytoplasmic proteins or organelles, entraps them into vesicles, and fuses them with lysosomes to form autophagic lysosomes that degrade the contents they entrap. Studies have shown that autophagy can scavenge waste within the cardiac system to achieve cellular homeostasis and organelle renewal (Wu et al., 2019). Under baseline conditions, autophagy plays a protective role in the structure and function of the heart, and can be activated under pressure to limit the aggravation of injury (Sciarretta et al., 2018). However, excessive and sustained activation of autophagy may cause heart damage under certain stress conditions such as reperfusion injury, starvation, diabetes, hypertension, and ischemia (Sciarretta et al., 2018; Shi et al., 2021). In pathological conditions, to fight the stress response, the heart will exhibit unrestrained autophagic activity, which promotes maladaptive cardiac remodeling and contributes to the development of cardiomyopathy (Porrello and Delbridge, 2009; Mei et al., 2015). However, the role of autophagy in UCM and the specific mechanisms involved are still unclear.

Hydrogen sulfide (H_2_S) is a gasotransmitter that is endogenously produced in the cardiovascular system by cystathionine gamma-lyase (CSE), which has shown great potential in treating cardiovascular diseases (CVDs) (Kolluru et al., 2015). There is increasing evidence that H_2_S regulates vascular tone and myocardial function in association with altered autophagy (Zhang et al., 2018; LaPenna et al., 2021). Studies have demonstrated that the protective effect of H_2_S against diabetes-induced myocardial fibrosis may be associated with attenuation of autophagy (Xiao et al., 2016). In smoking-induced cardiomyopathy, H_2_S administration can decrease autophagy (Zhou et al., 2014). However, the effects of H_2_S on UMC and its related mechanisms are still unknown.

Available evidence has demonstrated that mTOR activation is regulated by PI3K/PKB pathway activation, leading to inhibition of excessive autophagy and protection from cardiac hypertrophy (Kishore et al., 2015; Cheng et al., 2017). Thus, we hypothesized that H_2_S protects against UCM *via* regulation of PI3K/PKB/mTOR-mediated autophagy. This study designed and conducted a series of *in vitro* and *in vivo* experiments to verify this hypothesis.

## 2 Materials and methods

### 2.1 Animals

C57BL/6 mice aged 9 weeks were purchased from Beijing Vital River Laboratory Animal Technology Co., Ltd (License No. SCXK, 2016–0006, Beijing) and were fed adaptively for 1 week. Animals were housed in a clean degree laboratory with three mice per cage, under a controlled temperature of 24°C, relative humidity of 35–50%, with free access to food and water.

### 2.2 Mouse model of 5/6 nephrectomy-induced UCM, drug administration and experimental groups

**FIGURE 1:**
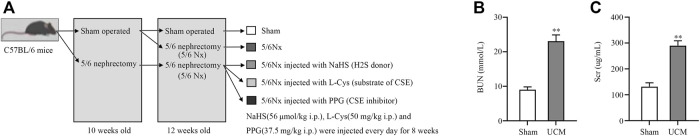
*In vivo* protocol and index of renal function in the Sham and uremic cardiomyopathy (UCM) groups. **(A)** Animal grouping and timeline for *in vivo* experiments. **(B)** Blood urea nitrogen concentrations in the Sham and UCM groups. **(C)** Serum creatinine concentrations in the Sham and UCM groups. Data are represented as Mean ± SEM. ^**^ *p* < 0.01 vs. Sham group.

Under isoflurane anesthesia, the mouse model of 5/6 nephrectomy was performed as previously described (Leelahavanichkul et al., 2010). In brief, a longitudinal incision of approximately 1 cm was made on the lateral skin below the angle of the left costal ridge in mouse. Subsequently, the muscle was cut layer by layer, the left kidney was exposed, the renal capsule was separated, and 2/3 of the upper and lower pole renal tissue of the left kidney was removed, and then a gelatin sponge was used to compress and stop bleeding. One week later, the right kidney was exposed in the same way and the pedicle was ligated with 3-0 non-absorbable sutures. The right kidney was removed after ligation. In the Sham group, the skin was cut under anesthesia to separate the renal capsule, but the kidney was not resected. Eight weeks after the operation, obvious renal dysfunction was observed in UCM mice (Figures 1B, C,) using serum biochemistry, as described in Section 2.3 below.

Male C57BL/6J mice were fed with normal chow diet and randomly divided into five groups (*n* = 6 per group): Sham, UCM, UCM + NaHS, UCM + L-cys, and UCM + PPG. The Sham group mice had a renal capsule isolated without kidney damage. The UCM group mice underwent 5/6 nephrectomy. The UCM + NaHS group mice had UCM and were intraperitoneally injected with NaHS (H_2_S donor, 56 μmol/kg body weight/day). The UCM + L-cys group mice had UCM and were intraperitoneally injected with L-cys (substrate of CSE, 50 mg/kg body weight/day). The UCM + PPG group mice had UCM and were intraperitoneally injected with PPG (CSE inhibitor, 37.5 mg/kg body weight/day) (Figure 1A). All procedures were performed in accordance with the guidelines set by the recommendations of the Guide for the Care and Use of Laboratory Animals of the National Institutes of Health and approved by the Experimental Animal Ethics Committee of Beijing Chao-Yang Hospital, Capital Medical University.

### 2.3 Measurement of serum creatinine and blood urea nitrogen

Mice were fasted for at least 8 h before blood was collected in anticoagulant tubes. Serum creatinine and blood urea nitrogen were measured using the sarcosine oxidase method (Solarbio, BC4910, Beijing, China) and visible spectrophotometry (Solarbio, BC1530, Beijing, China) respectively, according to the manufacturer’s protocol.

### 2.4 Measurement of H_2_S concentration

H_2_S concentrations in the plasma of all mice were measured using the zinc acetate reaction. The fresh blood was centrifuged at 500 g for 5 min, the plasma was immediately obtained, and rapidly added to the assay mixture. First, 100 μL of plasma was added into a test tube containing distilled water and 1% zinc acetate; thereafter, N, N-dimethyl-p-phenylenediamine dihydrochloride in 7.2 M HCl and FeCl_3_ in 1.2 M HCl were added. This mixture was incubated at room temperature for 20 min, after which 1 ml of 10% trichloroacetic acid was added to the solution and centrifuged at 4,000 g for 10 min. The optical absorbance of the supernatant was measured with a spectrometer at 670 nm. All samples were assayed repeatedly, and the H_2_S concentration in the solution was calculated against the calibration curve of the standard NaHS solution.

H_2_S concentrations in H9C2 cardiomyocytes were measured using the micro-method (Solarbio, BC 2055, Beijing, China), according to the manufacturer’s protocol.

### 2.5 Histopathology

Fresh mouse heart tissue was fixed in 4% paraformaldehyde solution, embedded in paraffin, and then cut into 4 μm thick slices. The cut slices were dewaxed in xylene, with degraded alcohol hydration. The slides were stained with hematoxylin and eosin (H&E) or Masson’s trichrome (Servicebio, G1005, Wuhan, China) according to the instructions.

For H&E staining, the degree of myocardial injury was scored according to the following criteria: 0, myocardial structure intact without injury; 1, mild myocardial interstitial edema and local necrosis; 2, extensive myocardial and interstitial edema accompanied by local medial necrosis; 3, severe small vessel injury and extensive myocardial necrosis, with a large number of inflammatory cell infiltrations and contraction band formation; and 4, severe diffuse myocardial necrosis and hemorrhage, massive small vessel injury, and formation of contraction band.

For Masson’s trichrome staining results, collagen fibers were blue. The volume fraction of collagen was calculated as the ratio of positive collagen blue area to the whole tissue area.

### 2.6 Electrocardiogram measurements

A high-frequency ultrasound device (Vevo 2100, VisualSonics, Toronto, Canada) was utilized for ultrasound imaging. During this process, mice were anesthetized with 2–3% isoflurane in the induction chamber and maintained by inhalation of 1.0–1.5% isoflurane with 100% oxygen mask. Measures across three cardiac cycles were averaged for all electrocardiogram measurements.

Measures from the left ventricle (LV) mid-papillary level in the parasternal long-axis view were conducted with 2D M-mode imaging, for: interventricular septum thicknesses at end-diastole and end-systole (IVSd and IVSs, respectively); LV internal diameters at end-diastole and end-systole (LVIDd, LVIDs); LV posterior wall thicknesses at end-diastole and end-systole (LVPWd and LVPWs); and stroke volume (SV), which was derived by the formula: EDV—ESV,

Where EDV and ESV are the end-diastolic and end-systolic volumes, respectively. LV systolic function was estimated by the ejection fraction (EF) derived from the formula: SV/EDV.

The area-length method (Zhang T. Y. et al., 2021) was used to calculate the LV mass, which was derived using the following formula: LV mass, g = 1.053 [(LVIDd + IVSd + LVPWd)^3^ − (LVIDd)^3^]; LV mass (corrected), g = 0.8 × LV mass.

Doppler measurements of mitral valve inflow velocities were recorded from an apical four-chamber view, with a cursor positioned at the tips of the mitral valve leaflets. Diastolic function was evaluated from measurements of early filling velocity (E), atrial filling velocity (A), and calculation of the ratio of the early to late diastolic mitral inflow velocities (E/A).

### 2.7 Cell culture and treatment and RNA infection

A sample of H9C2 cardiomyocytes was obtained from the China Cell Bank. These were cultured in Dulbecco’s modified Eagle’s medium (DMEM, 11966025, Gibco, NY 14072, United States) containing 10% (v/v) fetal bovine serum (FBS, 10099-141, Gibco, Grand Island, NY 14072, United States) and 1% (v/v) penicillin-streptomycin solution (15070063, Life Technologies, Carlsbad, Ontario, Canada) at 37°C in an incubator (MCO-5AC, Panasonic, Kyoto, Japan) with 5% CO_2_.

H9C2 cardiomyocytes were transiently transfected with CSE-specific siRNA (Beijing likely biotechnology, Beijing, China) using lipofectamine 2000 (Thermo Fisher Scientific, Waltham, United States), and the cells were subsequently cultured for 48 h.

### 2.8 Human blood sampling

Human serum samples were taken to stimulate H9C2 cardiomyocytes *in vitro*. These samples were collected from four healthy participants and nine patients with end-stage renal failure who underwent hemodialysis. Serum was collected before the start of the hemodialysis session. The mean age of the healthy participants was 58.50 ± 7.05 years, and none had a family history of diabetes, cardiovascular disease, or serious liver or kidney disease. The mean age of the hemodialysis patients was 58.89 ± 8.91 years, while the mean duration of hemodialysis therapy was 220.78 ± 31.46 months. Color doppler echocardiography was used to investigate enlargement of the heart. The study was conducted in accordance with the Declaration of Helsinki and approved by the Ethics Committee of Beijing Chao-Yang Hospital, Capital Medical University. Written informed consent was obtained from all participants.

### 2.9 Detection of cell viability

Cell Counting Kit-8 (CCK-8) assay (C0038, Beyotime Biotechnology, Shanghai, China) was used to assess H9C2 cell viability. Briefly, cells were cultivated in 96-well plates (2000 cells/well) and exposed to different concentrations of uremic serum and 25–200 μm NaHS in 10% uremic serum for 24 h. Then, 10 μL of the kit solution was mixed into the culture medium for 1 h. Absorbance was measured at 450 nm using a microplate reader (PerkinElmer EnSpire, MA, United States). Cell viability was expressed as a percentage of the control.

### 2.10 Western immunoblotting analysis

Mouse heart tissues and H9C2 cardiomyocytes were homogenized and thawed in radioimmunoprecipitation assay buffer (C1053, Applygen, Beijing, China) with protease inhibitors (04693116001, Roche, United States) and phosphatase inhibitors (04906837001). The protein concentration was determined using a BCA Protein Assay kit (23225; Pierce Company, Rockford, IL, United States). The samples were subjected to sodium dodecyl-sulfate polyacrylamide gel electrophoresis (SDS-PAGE) and subsequently transferred onto a polyvinylidene difluoride membrane (GE Healthcare, Buckinghamshire, United Kingdom). The membranes were blocked with 10% milk and incubated with primary antibodies at 4°C overnight. The primary antibodies used were CSE (1:1000, 12217-1-AP) and P62 (1:1000, 18420-1-AP) from Proteintech (Rosemont, IL 60018, United States); LC3 A/B (1:1000, 12741), P-p85 (Tyr458)/p55 (Tyr199)-PI3K (1:1,000, 4,228), PI3K (1:1,000, 4,257), P-Ser473-PKB (1:1,000, 4,060), PKB (1:1,000, 9272), mammalian target of rapamycin (mTOR, 1:1,000, 2983), P-Ser2448-mTOR (1:1,000, 5536) from Cell Signaling Technology (Danvers, MA 01923, United States); and GAPDH (1:5000, C1846) from Applygen (Beijing, China). Samples were treated with horseradish peroxidase-conjugated goat anti-mouse IgG or goat anti-rabbit IgG secondary antibodies (1:4,000, 31460, Thermo Fisher, Marietta, GA, 30006, United States) for 1 h at room temperature. Fusion FX (Vilber, France) and Enhanced Chemiluminescence kit (GE Healthcare, Chicago, Illinois, United States) were used to detect the signals. ImageJ software (National Institutes of Health, Bethesda, MD, United States) was used for quantitative Western blot analysis.

### 2.11 Statistical analysis

All cell experiments were performed at least three times, while all animal experiments were performed at least six times. GraphPad Prism software (version 8.0; GraphPad Software, La Jolla, United States) was used for statistical analysis. The measurement data are presented as the mean ± SEM, and statistical analyses were performed using one-way analysis of variance, followed by all pairwise multiple comparison procedures using the Bonferroni test. Categorical functional outcomes data, such as H&E staining, were expressed as quartiles, and the Mann-Whitney *U* test was used for group comparisons. Statistical significance was set at *p* < 0.05.

## 3 Results

### 3.1 H_2_S inhibited myocardial disarray and fibrosis in UCM mice

**FIGURE 2:**
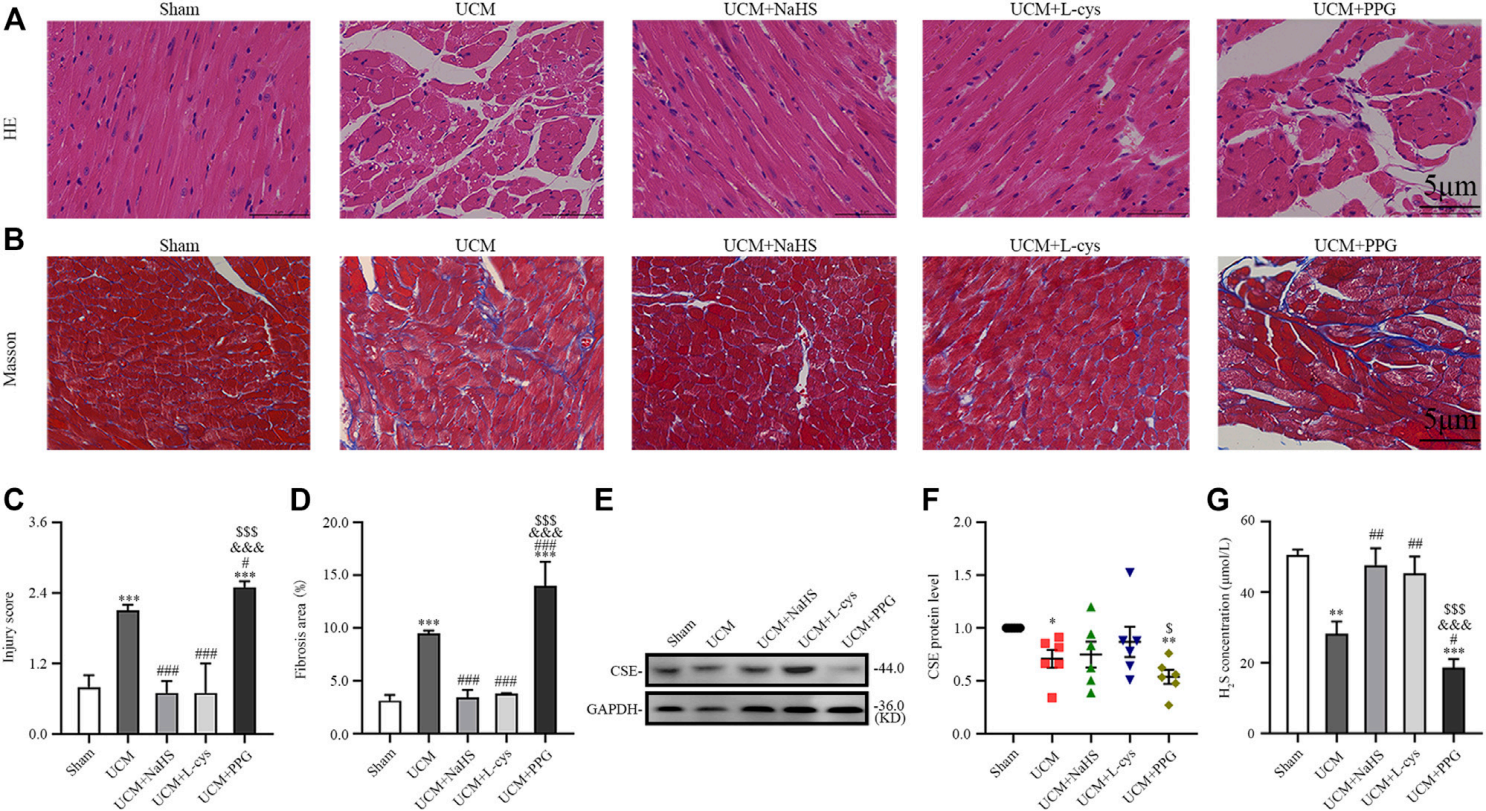
H_2_S treatment inhibit myocardial disarray and fibrosis in UCM mice, whereas H_2_S deficiency aggravates injuries. **(A)** Representative images for H&E staining of heart tissues. Scale bar = 5 μm. **(B)** Representative images of Masson’s trichrome staining of heart tissues. Scale bar = 5 μm. **(C)** Statistical testing results of injury level scoring, with *n* = 3 per group. **(D)** Statistical testing results of the fibrosis area, with *n* = 3 per group. **(E)** Representative Western blot images of CSE expressions in the heart tissue of the five groups of mice. **(F)** Quantification of CSE protein expressions in the in the heart tissue of the five groups of mice, with *n* = 6 per group. **(G)** Quantification of H_2_S concentration in the heart tissue of the five groups of mice, with *n* = 6 per group. Data are represented as Mean ± SEM. ^*^ *p* < 0.05, ^**^ *p* < 0.01, ^***^ *p* < 0.001 vs. Sham group; ^#^ *p* < 0.05, ^##^ *p* < 0.01, ^###^ *p* < 0.001 vs. UCM group; ^&&&^ *p* < 0.001 vs. UCM + NaHS group; ^$^ *p* < 0.05, ^$$$^ *p* < 0.001 vs. UCM + L-cys group.

To investigate the role of H_2_S in UCM, we first examined CSE expression in the heart of each group of mice. Eight weeks after the operation, Western blotting results showed that the expression of CSE protein in the heart of UCM mice was decreased compared with mice in the Sham group. Intraperitoneal injection of NaHS and L-Cys had no effect on the protein expression of CSE in the heart of UCM mice, but the expression of CSE was further decreased after PPG treatment (Figures 2E, F). Additionally, we measured the H_2_S concentration in mouse plasma. Compared with mice in the Sham group, mice in the UCM group showed significantly lower H_2_S levels. Intraperitoneal injection of NaHS and L-Cys reduced the decrease of H_2_S levels, whereas PPG administration enhanced the decrease of H_2_S levels in mice in the UCM group (Figure 2G).

Morphologically, H&E results suggested that compared with the mice in the Sham group, mice in the UCM group had obvious myocardial disorder (Figures 2A,C). In contrast, the above abnormalities could be reversed by NaHS and L-Cys treatment and further aggravated after PPG intraperitoneal injection (Figures 2A,C). Masson’s staining also indicated that the level of cardiac fibrosis induced by 5/6 nephrectomy was significantly decreased when NaHS and L-Cys treatments were applied. However, the fibrosis level caused by 5/6 nephrectomy surgery in mice hearts was further aggravated after PPG treatment (Figures 2B,D).

### 3.2 H_2_S improved LVH and diastolic dysfunction in UCM mice

**FIGURE 3:**
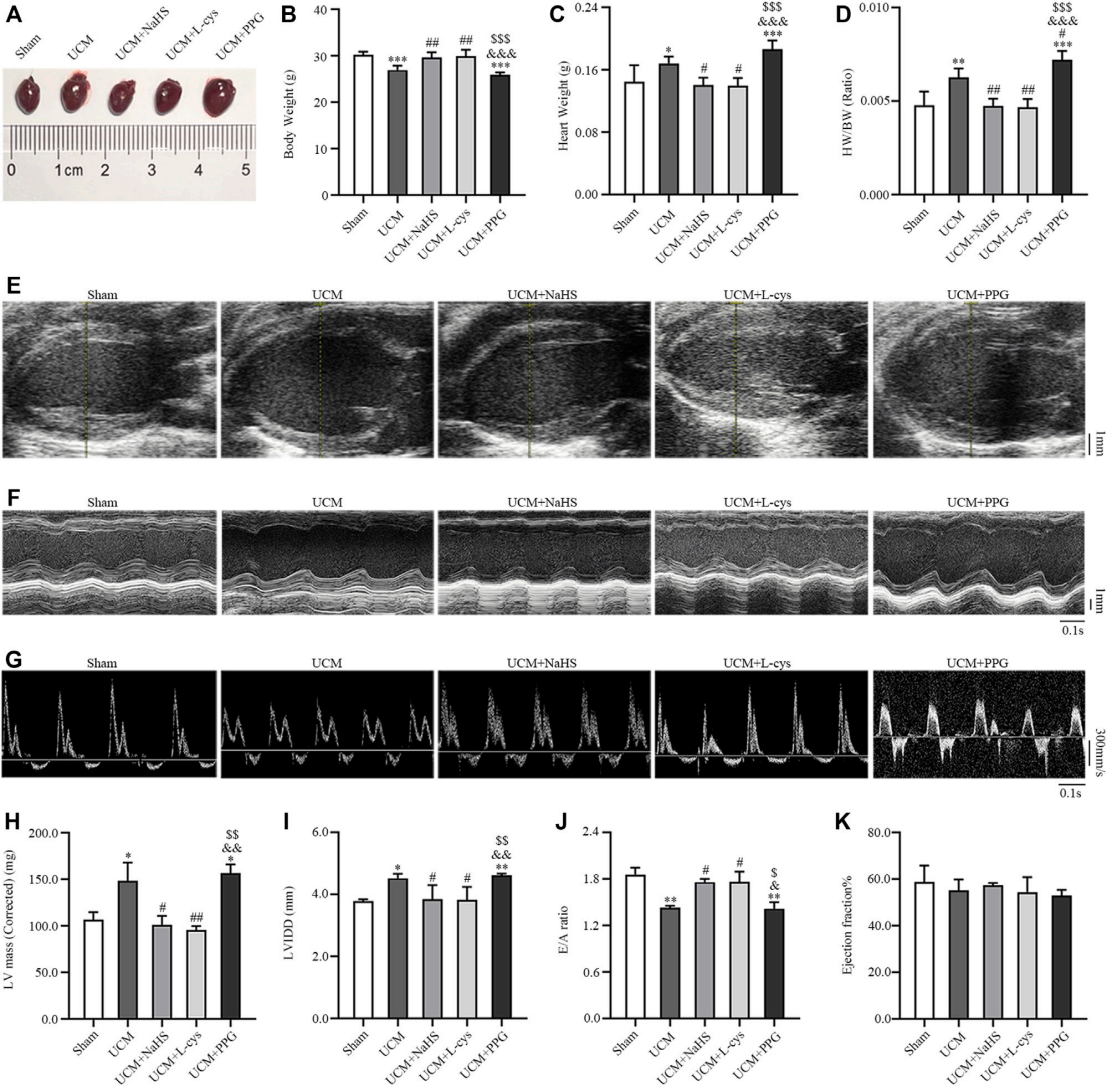
NaHS and L-cys treatment improved left ventricular hypertrophy (LVH) and diastolic dysfunction in UCM mice. **(A)** Representative images of heart tissues in the five groups of mice. **(B–D)** H_2_S treatment reduces heart weight and heart weight/body weight. *n* = 6 per group. **(E–G)** Representative echocardiographic images in the five groups of mice. **(E)** Two-dimensional echocardiograms showing left ventricular long-axis views, with scale bar in mm on right. **(F)** M-mode echocardiograms showing left ventricular dimensions, with scale bar in mm on right and time stamp in seconds at the bottom. **(G)** Pulse-wave Doppler echocardiograms depicting mitral inflow velocities, with scale bar in mm on right and time stamp in seconds at bottom. **(H–K)** H_2_S treatment improves LVH and diastolic dysfunction in UCM mice. *n* = 3 per group. E/A: the ratio of the early to late diastolic mitral inflow velocities; LVIDd: LV internal diameters at end-diastole; HW/BW: heart weight/body weight. Data are represented as Mean ± SEM. ^*^ *p* < 0.05, ^**^ *p* < 0.01, ^***^ *p* < 0.001 vs Sham group; ^#^ *p* < 0.05, ^##^ *p* < 0.01 vs. UCM group; ^&^ *p* < 0.05, ^&&^ *p* < 0.01, ^&&&^ *p* < 0.001 vs. UCM + NaHS group; ^$^ *p* < 0.05, ^$$^ *p* < 0.01, ^$$$^ *p* < 0.001 vs. UCM + L-cys group.

We investigated whether H_2_S improves the prognosis of UCM *in vivo*. Compared with mice in the Sham group, the heart weight, heart weight/body weight (HW/BW), cardiac structural parameters LV mass and LVIDd were significantly higher, while body weight and diastolic functional values E/A were significantly lower in mice in the UCM group (Figures 3B–D, H–J). These abnormalities were largely reversed by NaHS and L-Cys treatment (Figures 3B–D, H–J). No significant differences were observed between groups for cardiac systolic ejection fractions (Figure 3K). Taken together, these studies identified that 5/6 nephrectomy-induced uremia can lead to cardiac dysfunction, including increased heart weight, LVH and diastolic dysfunction, which can be supplemented with H_2_S torsion.

### 3.3 H_2_S treatment inhibited PI3K-PKB-mTOR-activated autophagy in UCM mice

**FIGURE 4:**
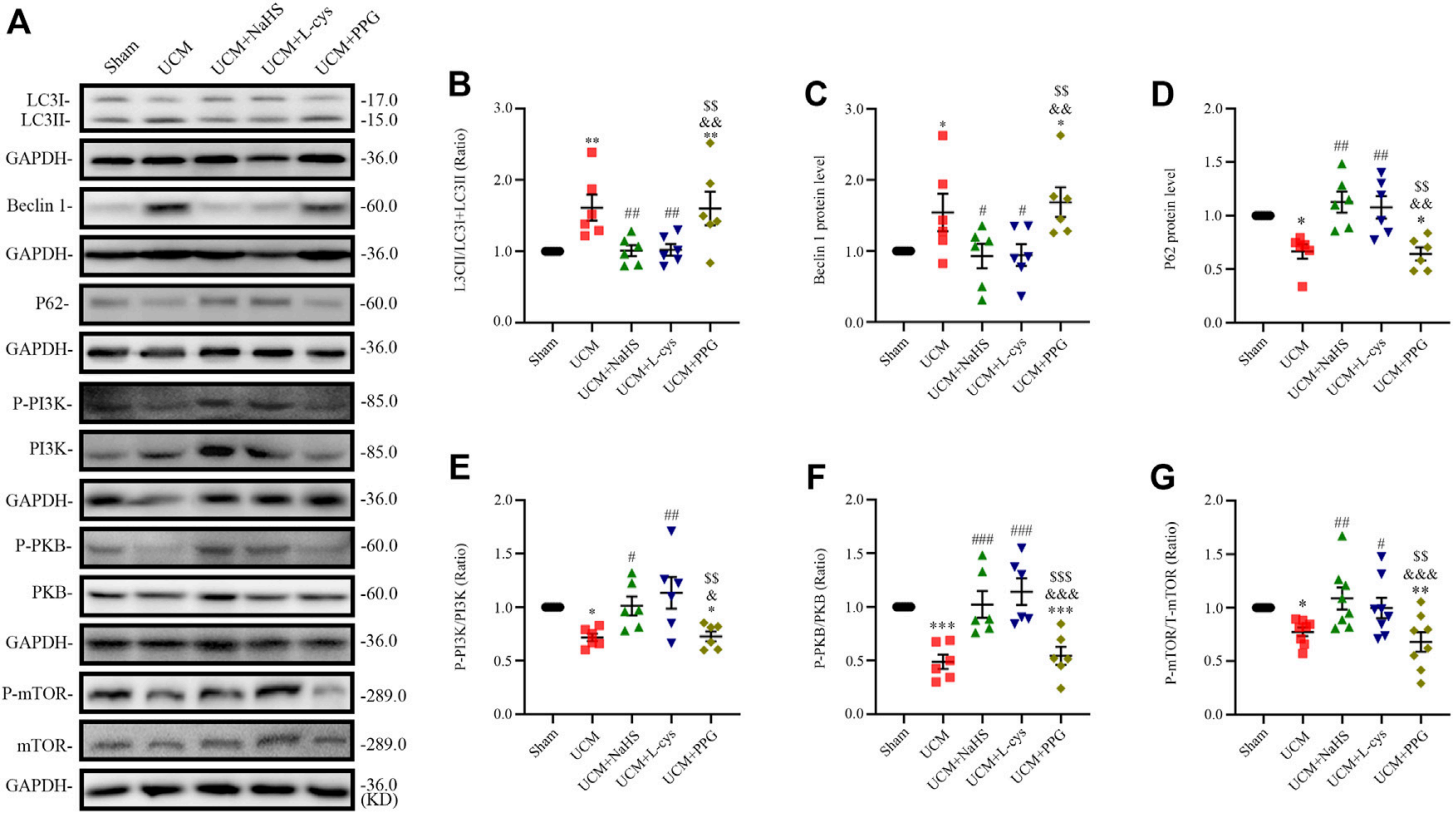
NaHS and L-cys treatment inhibit PI3K-PKB-mTOR-activated autophagy in UCM mice. **(A)** Representative Western blot images of LC3, becline-1, P62, p-PI3K/PI3K, p-PKB/PKB and p-mTOR/mTOR in the heart tissue of the five groups of mice. **(B–D)** Quantification of the LC3II/LC3 (I + II) ratio, becline-1 protein levels, and p62 protein levels in the heart tissue of the five groups of mice, with *n* = 6 per group. **(E–G)** Quantification of the p-PI3K/PI3K (*n* = 6 per group), p-PKB/PKB (*n* = 6 per group) and P-mTOR/mTOR (*n* = 8 per group) levels in the heart tissue of the five groups of mice. Data are represented as Mean ± SEM. ^*^ *p* < 0.05, ^**^ *p* < 0.01, ^***^ *p* < 0.01 vs. Sham group; ^#^ *p* < 0.05, ^##^ *p* < 0.01, ^###^ *p* < 0.001 vs. UCM group; ^&^ *p* < 0.05, ^&&^ *p* < 0.01, ^&&&^ *p* < 0.001 vs. UCM + NaHS group; ^$$^ *p* < 0.01, ^$$$^ *p* < 0.001 vs. UCM + L-cys group.

To determine whether 5/6 nephrectomy-induced UCM could regulate autophagy, we detected the expression of autophagy-related molecules in mouse heart tissue. We observed that the LC3II/LC3(I + II) ratio, becline-1 protein levels, and p62 degradation significantly increased in UCM mice compared to the Sham group (Figures 4A–D), suggesting that UCM may lead to overactive autophagy. However, intraperitoneal injection of NaHS and L-Cys decreased the LC3II/LC3 (I + II) ratio, becline-1 protein levels, and p62 degradation. The same measures increased after PPG treatment, indicating that H_2_S attenuated overactive autophagy in the heart tissue of 5/6 nephrectomy-induced UCM mice (Figures 4A–D).

Autophagy is mediated by several pathways, including the PI3K/PKB/mTOR pathway. We further verified the effects of H_2_S on the PI3K-PKB-mTOR signaling pathway after 5/6 nephrectomy in mice. Western blotting analysis showed that protein expression levels of phosphorylated-PI3K (p-PI3K), p-PKB, and p-mTOR were significantly lower in the UCM and UCM + PPG groups, and we observed that the addition of NaHS and L-cys significantly increased the phosphorylation levels of PKB, PI3K, and mTOR (Figures 4A, E–G). These results suggest that H_2_S inhibits excessive autophagy in the heart tissue by activating the PI3K-PKB-mTOR pathway.

### 3.4 Serum uremia induces overactive autophagy in H9C2 cardiomyocytes

**FIGURE 5:**
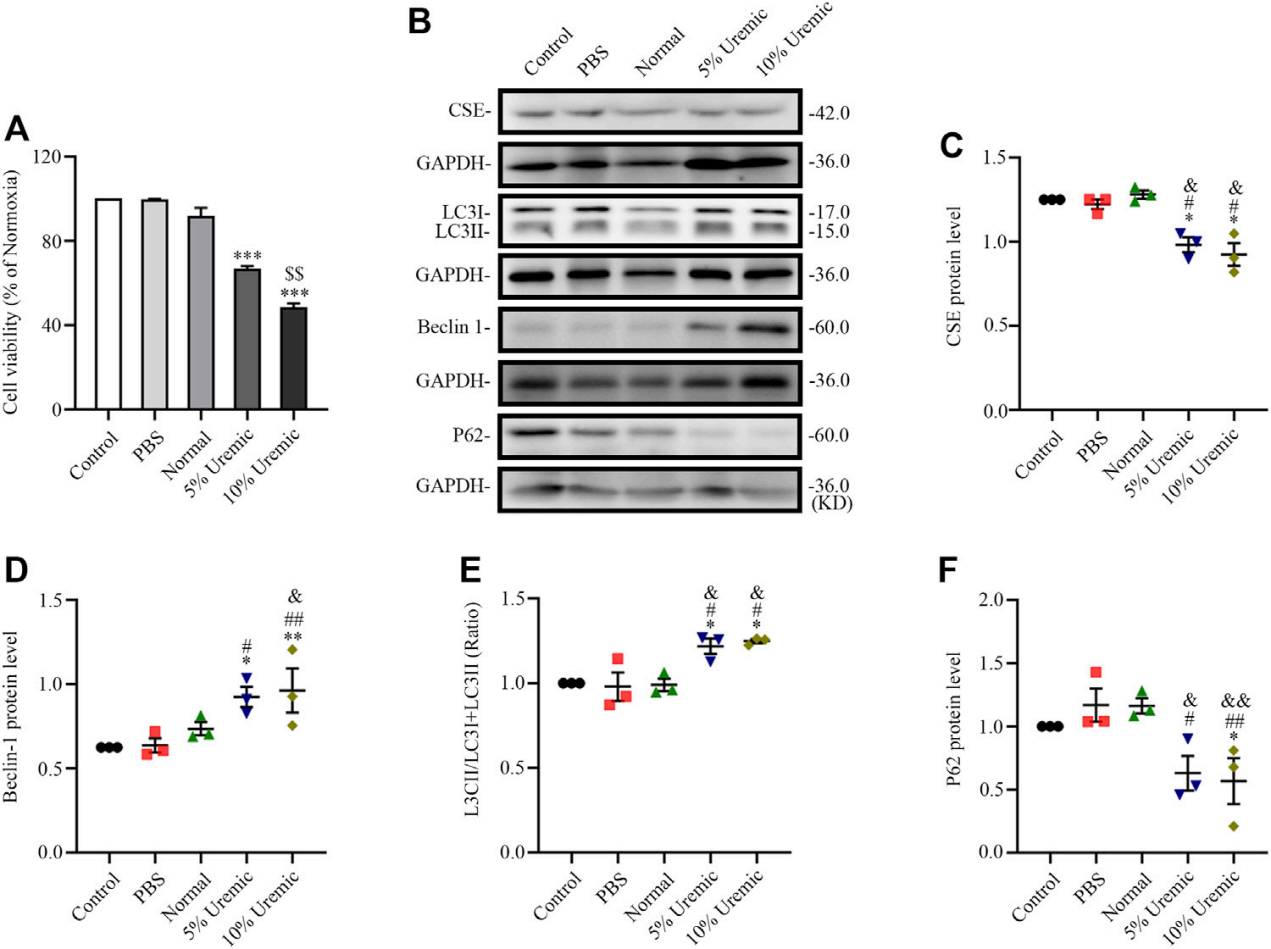
Effects of serum uremia on the expressions of cystathionine gamma-lyase (CSE) protein and autophagy-associated protein. **(A)** Cell viability was measured using the CCK-8 assay, with *n* = 3 per group. **(B)** Representative Western blot images of CSE, LC3, becline-1, and P62 in the five groups of H9C2 cardiomyocytes **(C–F)** Quantification of the CSE protein levels, LC3II/LC3 (I + II) ratio, becline-1 protein levels, and p62 protein levels in the five groups of H9C2 cardiomyocytes. *n* = 3 per group. Data are represented as Mean ± SEM. ^*^ *p* < 0.05, ^**^ *p* < 0.01, ^***^ *p* < 0.001 vs. control group; ^#^ *p* < 0.05, ^##^ *p* < 0.01 vs. PBS group; ^&^ *p* < 0.05, ^&&^ *p* < 0.01 vs normal serum group; ^$$^ *p* < 0.01 vs. 5% uremic serum group.

The *in vitro* experiment was divided into four parts. In the first part, H9C2 cardiomyocytes were treated with normal serum and different concentrations of uremic serum, respectively. The results showed that, following exposure to uremic serum for 24 h, the H9C2 cardiomyocytes showed significantly decreased viability and lower levels of CSE expression (Figures 5A–C). Western blotting showed the protein expression of LC3II/LC3(I + II) ratio, becline-1 protein levels, and p62 degradation were higher in cardiomyocytes treated with uremic serum than in those treated with PBS or normal serum (Figures 5B, D–F). These results indicate that serum uremia may injure H9C2 cardiomyocytes *via* inducing overactive autophagy. In addition, H9C2 cells exposed to 10% uremic serum had higher cytotoxicity, becline-1 protein levels and p62 degradation than those exposed to 5% uremic serum (Figures 5A–C, F). Thus, 10% uremic serum was selected to mimic the uremic environment *in vitro*.

### 3.5 Endogenous H_2_S deficiency exacerbates uremic serum-induced overactive autophagy in H9C2 cardiomyocytes

**FIGURE 6:**
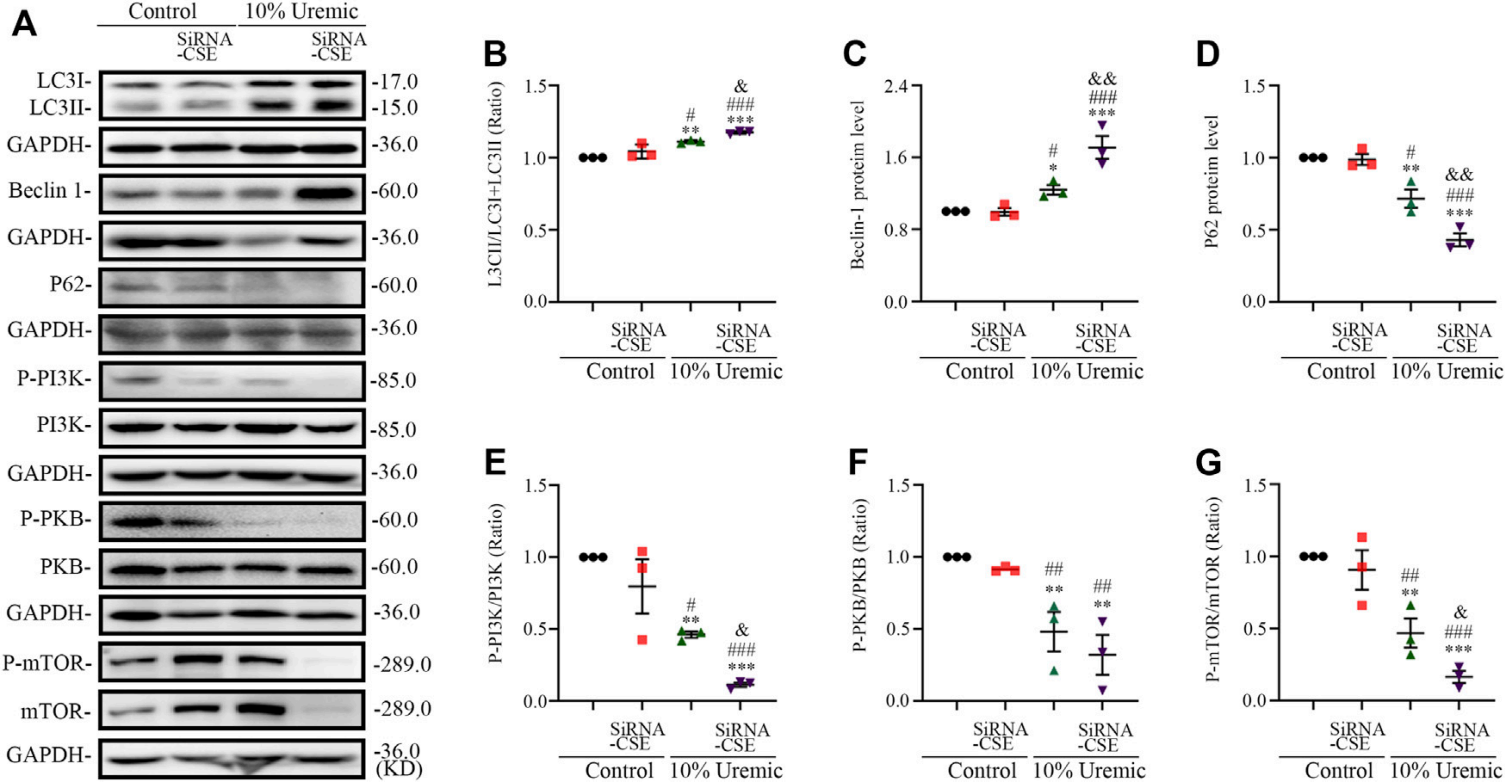
Endogenous H_2_S deficiency exacerbates uremic serum-induced overactive autophagy in H9C2 cardiomyocytes. **(A)** Representative Western blot images of LC3, becline-1, P62, p-PI3K/PI3K, p-PKB/PKB, and p-mTOR/mTOR in four groups of H9C2 cardiomyocytes. **(B–D)** Quantification of the LC3II/LC3 (I + II) ratio, becline-1 protein levels, and p62 protein levels in the four groups of H9C2 cardiomyocytes, with *n* = 3 per group. **(E–G)** Quantification of p-PI3K/PI3K, p-PKB/PKB, and p-mTOR/mTOR in four groups of H9C2 cardiomyocytes, with *n* = 3 per group. Data are represented as Mean ± SEM. ^*^ *p* < 0.05, ^**^ *p* < 0.01, ^***^ *p* < 0.001 vs. control group; ^#^ *p* < 0.05, ^##^ *p* < 0.01, ^###^ *p* < 0.001 vs. 10% control + SiRNA-CSE group; ^&^ *p* < 0.05, ^&&^ *p* < 0.01 vs. 10% uremic serum + SiRNA-CSE group.

In the second phase of *in vitro* experiments, we attenuated the expression of CSE using siRNA transfection. The expression levels of autophagy-related proteins (LC3-II, Beclin1, and p62) were quantified by Western blotting. We observed that the LC3II/LC3(I + II) ratio, becline-1 protein levels, and p62 degradation significantly increased in the 10% uremic serum + SiRNA-CSE group, compared to the control and 10% uremic serum groups (Figures 6A–D). Western blot analysis showed that the protein expression levels of phosphorylated-PI3K (p-PI3K), phosphorylated-PKB (p-PKB), and p-mTOR were significantly lower in the 10% uremic toxin + SiRNA group than in the control and 10% uremic serum groups (Figures 6A, E–G). These results indicated that endogenous H_2_S deficiency exacerbates excessive autophagy *via* the PI3K-PKB-mTOR pathway in H9C2 cardiomyocytes exposed to 10% uremic serum.

### 3.6 H_2_S inhibited serum uremia-induced overactive autophagy in H9C2 cardiomyocytes

**FIGURE 7:**
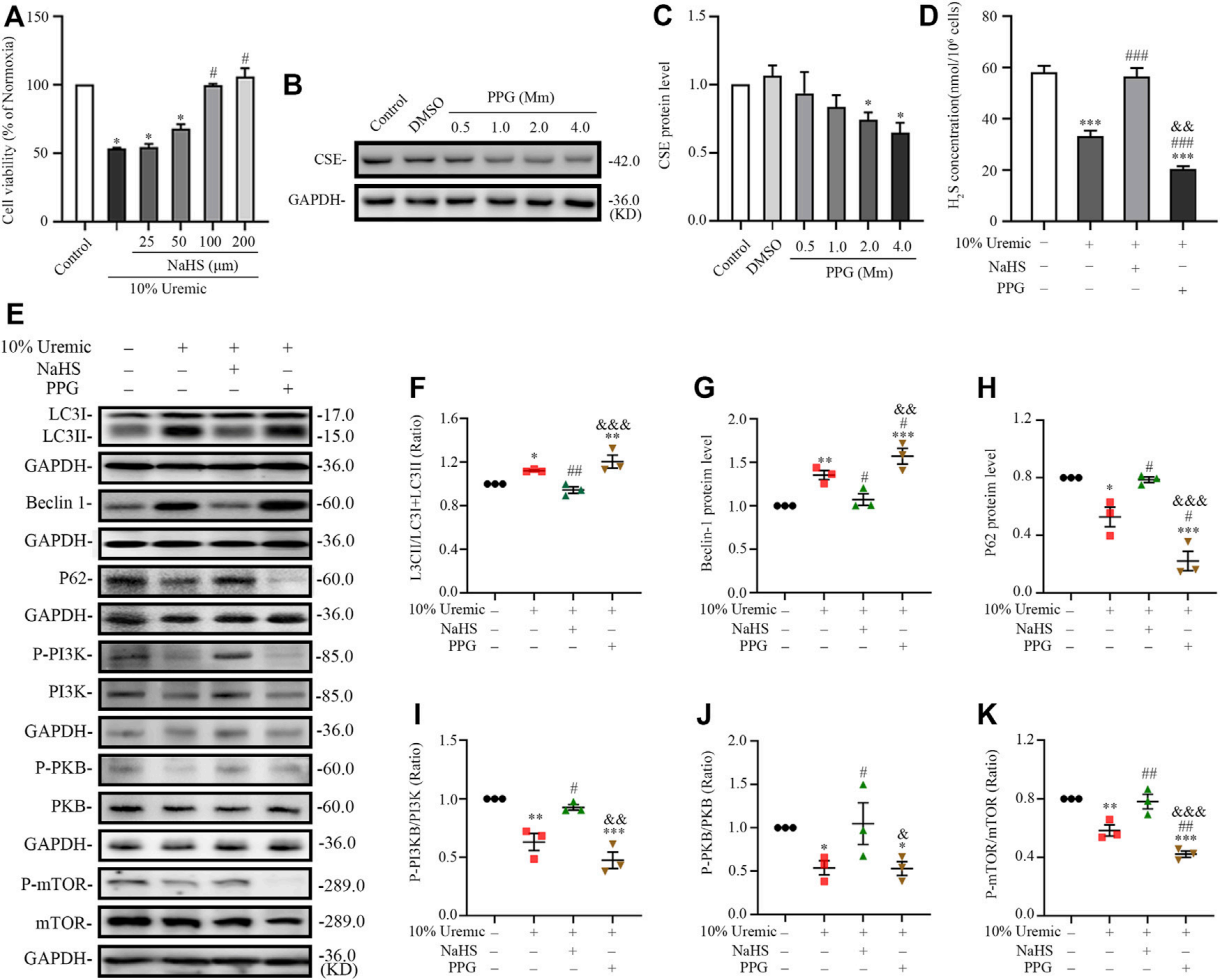
NaHS inhibits uremic serum-induced overactive autophagy in H9C2 cardiomyocytes, whereas deficiency of H_2_S further activates these effects. **(A)** Cell viability is measured using the CCK-8 assay, with *n* = 3 per group. **(B)** Representative Western blot images of inhibition of PPG on CSE protein levels in H9C2 cardiomyocytes. **(C)** Quantification of the CSE protein levels in the different concentrations of PPG in H9C2 cardiomyocytes, with *n* = 5 per group. **(D)** Quantification of H_2_S concentration in four groups of H9C2 cardiomyocytes, with *n* = 3 per group. **(E)** Representative Western blot images of LC3, becline-1, P62 p-PI3K/PI3K, p-PKB/PKB, and p-mTOR/mTOR in four groups of H9C2 cardiomyocytes. **(F–H)** Quantification of the LC3II/LC3(I + II) ratio, becline-1 protein levels, and p62 protein levels in the four groups of H9C2 cardiomyocytes, with *n* = 3 per group. **(I–K)** Quantification of the p-PI3K/PI3K, p-PKB/PKB, and p-mTOR/mTOR in four groups of H9C2 cardiomyocytes, with *n* = 3 per group. Data are represented as Mean ± SEM. ^*^ *p* < 0.05, ^**^ *p* < 0.01, ^***^ *p* < 0.001 vs. control group; ^#^ *p* < 0.05, ^##^ *p* < 0.01, ^###^ *p* < 0.001 vs. 10% uremic serum group; ^&^ *p* < 0.05, ^&&^ *p* < 0.01, ^&&&^ *p* < 0.001 vs. 10% uremic serum + NaHS group.

In the third phase of *in vitro* experiments, cardiomyocytes were treated with NaHS or PPG in 10% uremic serum. Cell viability was measured using the CCK-8 assay. The expression levels of CSE and autophagy-related proteins (LC3-II, Beclin1, and p62) were quantified by Western blotting. The viability of H9C2 cardiomyocytes pretreated with 100 μm NaHS for 30 min before exposure to serum uremia was significantly increased, indicating that NaHS can significantly reduce the cytotoxicity induced by serum uremia (Figure 7A). Thus, 100 µm NaHS was selected for the pretreatment of cardiomyocytes exposed to 10% uremic toxin for 30 min. Western blotting showed that H9C2 cardiomyocytes treatment with 2.0 mm PPG for 24 h significantly attenuated the expression of CSE protein (Figures 7B,C). We further measured the H_2_S concentration in H9C2 cardiomyocytes and found that the H_2_S level significantly decreased in the 10% uremic serum group compared to the control group. Pre-treatment with NaHS increased the H_2_S level, and the level decreased after treatment with 2.0 mm PPG for 24 h (Figure 7D). We observed that the LC3II/LC3(I + II) ratio, becline-1 protein levels, and p62 degradation significantly increased in the 10% uremic serum group compared to the control group (Figures 7E–H). However, pre-treatment with NaHS decreased the LC3II/LC3 (I + II) ratio, becline-1 protein levels, and p62 degradation, and increased after PPG treatment (Figures 7E–H). Western blot analysis showed that the protein expression levels of phosphorylated-PI3K (p-PI3K), phosphorylated-PKB (p-PKB), and p-mTOR were significantly lower in the 10% uremic toxin group than in the control group (Figures 7D,I–K). We observed that pre-treatment with NaHS significantly increased the phosphorylation levels of PKB, PI3K, and mTOR (Figures 7D,I–K). These results indicated that NaHS treatment suppressed excessive autophagy *via* the PI3K-PKB-mTOR pathway in H9C2 cardiomyocytes exposed to 10% uremic serum.

### 3.7 H_2_S induces PKB and mTOR phosphorylation in H9C2 cardiomyocytes stimulated by serum uremia

**FIGURE 8:**
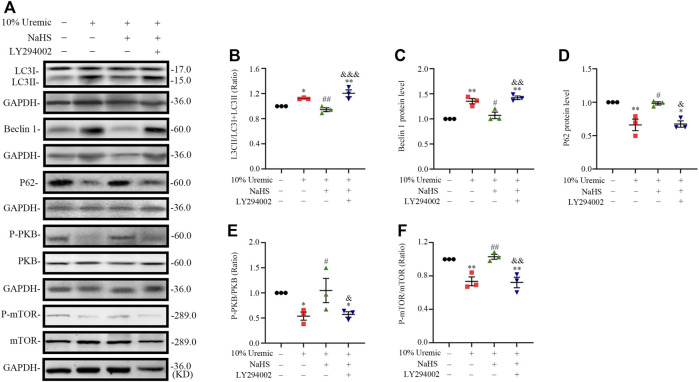
NaHS induces PKB and mTOR phosphorylation *via* PI3K/PKB pathway in H9C2 cardiomyocytes stimulated by uremic serum. **(A)** Representative Western blot images of LC3, becline-1, P62, p-PKB/PKB and p-mTOR/mTOR in four groups of H9C2 cardiomyocytes. **(B–D)** Quantification of the LC3II/LC3 (I + II) ratio, becline-1 protein levels, and p62 protein levels in the four groups of H9C2 cardiomyocytes, with *n* = 3 per group. **(E,F)** Quantification of the p-PKB/PKB and p-mTOR/mTOR in four groups of H9C2 cardiomyocytes, with *n* = 3 per group. Data are represented as Mean ± SEM. ^*^ *p* < 0.05, ^**^ *p* < 0.01 vs. control group; ^#^ *p* < 0.05, ^##^ *p* < 0.01 vs. 10% serum uremia group; ^&^ *p* < 0.05, ^&&^ *p* < 0.01, ^&&&^ *p* < 0.001 vs. 10% serum uremia + NaHS group.

In the fourth phase of *in vitro* experiments, H9C2 cardiomyocytes were treated with the PI3K inhibitor LY294002 prior to exposure to NaHS and 10% uremic serum, to investigate the role of the PI3K/PKB/mTOR pathway in the protective effects of H_2_S. We observed that pretreatment with NaHS decreased the LC3II/LC3 (I + II) ratio, becline-1 protein levels, and p62 degradation in 10% uremic serum. However, these protective effects of NaHS were blocked by LY294002 (Figures 8A–D). LY294002 also eliminated the stimulation of p-PKB and p-mTOR in the presence of NaHS but elicited no effect on the expression levels of total PKB and mTOR (Figures 8A, E, F). These results further suggest that the PI3K/PKB/mTOR pathway is involved in the protective effects of H_2_S.

**FIGURE 9:**
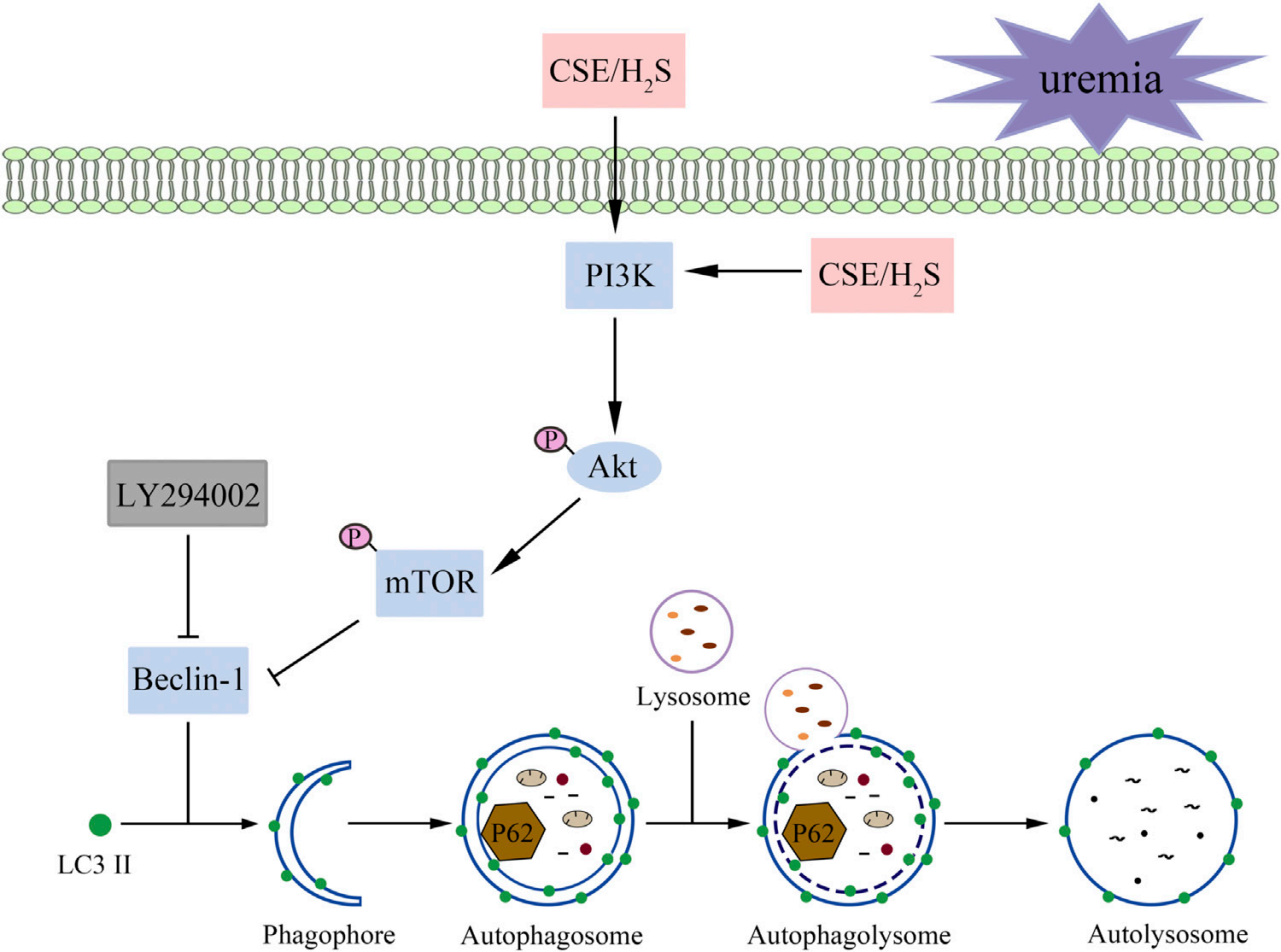
A proposed model illustrating the therapeutic effect of H_2_S on UCM. Uremia inhibits the phosphorylation of PI3K in the heart and results in the heart dysfunction of UCM mice, while H_2_S increases the phosphorylation levels of PKB, PI3K, and mTOR and attenuates overactive autophagy. In summary, H_2_S alleviates uremic cardiomyopathy by regulating PI3K/PKB/mTOR-mediated autophagy in 5/6 nephrectomy mice.

These results suggest that H_2_S increases the phosphorylation levels of PKB, PI3K, and mTOR and attenuates overactive autophagy in the hearts of UCM mice and serum uremia-treated cardiomyocytes. Inhibition of p-PI3K eliminated the protective effect of NaHS (Figure 9).

## 4 Discussion

UCM is a term used to describe patients with significant decline in renal function associated with cardiac structural and functional changes, which is almost ubiquitous in patients with end-stage renal disease (Wang et al., 2017; Sodhi et al., 2020). UCM most commonly manifests as LVH with preserved ejection fraction and predisposes the heart to further cardiovascular complications in CKD (Bao et al., 2020). The severity and persistence of LVH in CKD patients are closely related to acute cardiovascular events (such as sudden cardiac death, arrhythmia and acute myocardial infarction) and death risk (Husain-Syed et al., 2015; Duni et al., 2017). The pathogenesis of uremic cardiomyopathy is multifactorial. The current treatment can improve the outcome, but not provide a cure (Lekawanvijit, 2018).

Autophagy is a key pathway of programmed cell death that helps maintain cardiac homeostasis at the basic level (Abdellatif et al., 2020). However, abnormal autophagy is associated with the development and progression of heart disease (Shi et al., 2021). Some studies have shown that autophagy is generally decreased in aging hearts, and murine autophagy loss-of-function models develop exacerbated cardiac dysfunction (Shirakabe et al., 2016). While other studies have demonstrated that activation of autophagy has adverse effects on the heart, such as reperfusion injury, doxorubicin cardiomyopathy, and diabetic cardiomyopathy (Bartlett et al., 2017; Du et al., 2020; Zhang M. et al., 2021). Mialet-Perez et al. found that overactive autophagy promotes myocardial apoptosis and fibrosis and induces the transition from myocardial hypertrophy to heart failure (Mialet-Perez and Vindis, 2017). These inconsistent conclusions might be due to the different models used. Thus, there is an urgent need to understand the molecular mechanisms of UCM and identify drugs that are effective in treating UCM. In 5/6 nephrectomy mice, electrocardiogram showed that UCM developed cardiac alterations similar to those in patients with CKD-induced HFpEF. H&E and Masson’s staining indicated that mice in the UCM group exhibited significant myocardial disarray and fibrosis, respectively. Western blotting demonstrated elevated levels of autophagy-associated proteins in mice in the UCM group. We also found that serum uremia increased the expression of autophagy-associated proteins *in vitro*. These results suggest that activation of autophagy aggravates heart injury in 5/6 nephrectomy mice.

H_2_S is a newly discovered third gas signaling molecule after nitric oxide and carbon monoxide and has many biological effects in the cardiovascular system (Luo et al., 2020). Cystathionine β-synthase (CBS), CSE, and 3-mercaptopyruvate sulfurtransferase (3-MST) are the three key enzymes for the production of H_2_S in mammals (Bhatia and Gaddam, 2021). CSE is the major H_2_S -producing enzyme in the cardiovascular system and has recently been shown to be expressed in the liver, lungs, and kidneys (Wang, 2012). The present study demonstrated that CSE protein levels are decreased in the HFpEF phase of UCM in mice, and intraperitoneal injection of PPG resulted in a further decrease. We also found that intraperitoneal injection of NaHS and L-Cys markedly ameliorated myocardial disarray and fibrosis levels, LVH, and dysfunction of UCM in mice; however, intraperitoneal injection of PPG aggravated these injuries. These results indicate that endogenous H_2_S reduction and exogenous H_2_S supplementation can inhibit UCM development. Subsequently, *in vitro*, we found that NaHS pre-treatment attenuated the serum uremia-induced decrease in cardiomyocyte viability. We attenuated the expression of CSE using siRNA transfection and found endogenous H_2_S deficiency exacerbates excessive autophagy. Thus, these data showed that H_2_S could alleviate the development of UCM *in vivo* and *in vitro*. Furthermore, we found, by examining autophagy marker proteins (Beclin-1, LC3-II, and beclin-1) in the hearts of mice that underwent 5/6 nephrectomy and serum uremia-treated H9C2 cardiomyocytes, that supplementation with NaHS or L-Cys could decrease excessive autophagy activity. Consequently, we supposed that the protective effect of H_2_S on UCM was achieved by downregulating autophagy activation.

There are various signaling pathways regulating autophagy, PI3K/PKB pathway is one of the classical pathways regulating autophagy (Shao et al., 2016). The PI3K/PKB pathway plays an essential role in regulating cell growth and survival, and some studies have shown that this pathway can also mediate the survival of myocardial cell in many situations (Wang et al., 2015). Furthermore, mTOR can be phosphorylated as a downstream target of PI3K/PKB pathway (Shao et al., 2016). Previous studies have shown that mTOR activation is closely related to autophagy, and its activation may inhibit autophagy activity (Ahumada-Castro et al., 2019). Nie et al. demonstrated that H_2_S intervention in myocardial fibrosis is related to the inhibition of autophagy overactivation by upregulating of the PI3K/PKB/mTOR signal pathway (Nie et al., 2021). Xu et al. found that NaHS restores mitochondrial function and inhibits autophagy by activating the PI3K/PKB/mTOR signaling pathway to improve functional recovery after traumatic brain injury (Xu et al., 2018). The present study supports these previous findings, by demonstrating that supplementation with NaHS or L-Cys significantly increased the phosphorylation level of PI3K, PKB, and mTOR and decreased the autophagy-related protein expression levels (Beclin-1, LC3II). LY294002, as a PI3K specific inhibitor, significantly reversed the effects of NaHS in serum uremia-treated H9C2 cardiomyocytes. These results suggest that H_2_S may play a protective role in UCM by activating PI3K/PKB/mTOR pathway to inhibit excessive autophagy.

This study has several limitations. First, we did not use gene knockout or transgenic mice, but used pharmacological agonists or inhibitors which were selective. Second, we did not explore the dose gradient of L-Cys and NaHS injected intraperitoneally, but determined dose of the pharmacological agents from previous studies. Different doses may lead to different results, and future research should focus on addressing these important issues.

## 5 Conclusion

In summary, our study demonstrated for the first time that H_2_S has protective effects in UCM, which might be explained by the inhibition of autophagy *via* the activation of the PI3K/PKB/mTOR signaling pathway. These results support the view that H_2_S may be a potential therapeutic molecule to treat or prevent the development of UCM. It also increases the possibility of H_2_S as an anti-UCM drug for clinical use.

## Data Availability

The original contributions presented in the study are included in the article/Supplementary Material, further inquiries can be directed to the corresponding author.
