# Supplementary figures and images for: Hydrogen sulfide alleviates uremic cardiomyopathy by regulating PI3K/PKB/mTOR-mediated overactive autophagy in 5/6 nephrectomy mice
Source: Front Pharmacol. 2022 Dec 15;13:1027597. doi: 10.3389/fphar.2022.1027597 (PMC9797717; doi:10.3389/fphar.2022.1027597)

1. Representative Echocardiography examination images (Fig3)

Sham

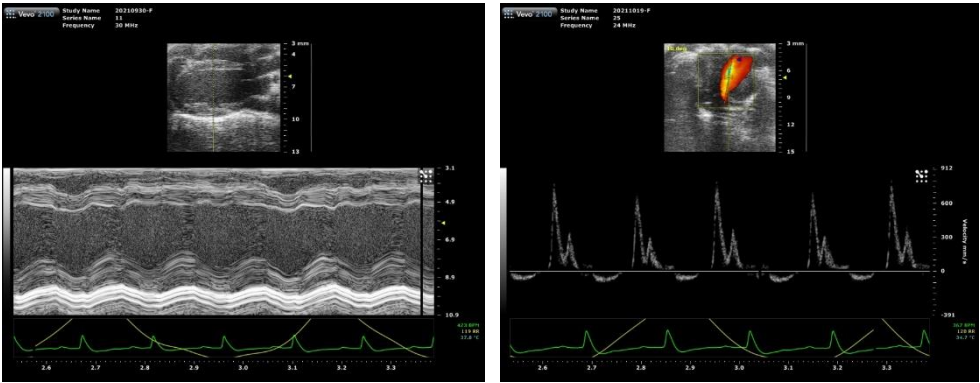

UCM

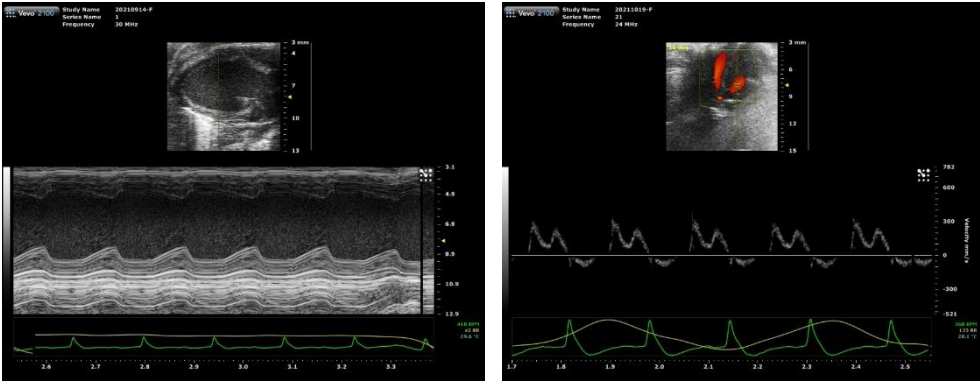

NaHS

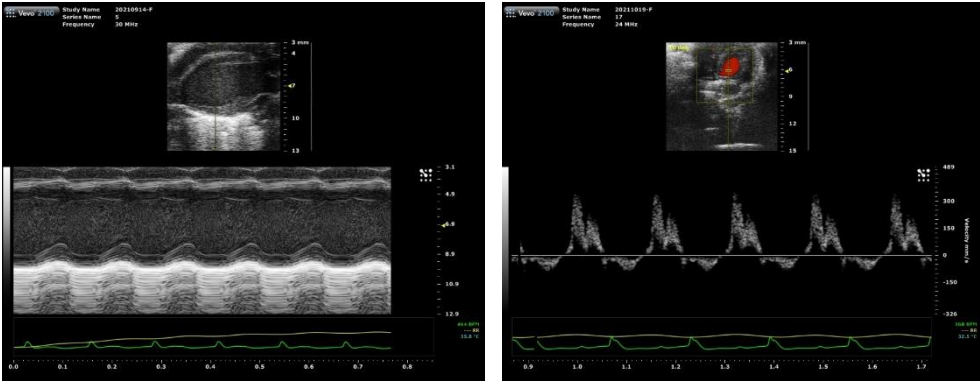

L-cys

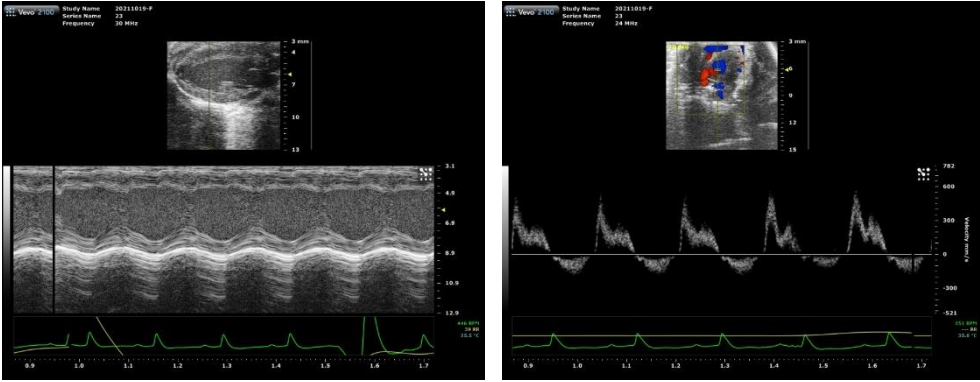

PPG

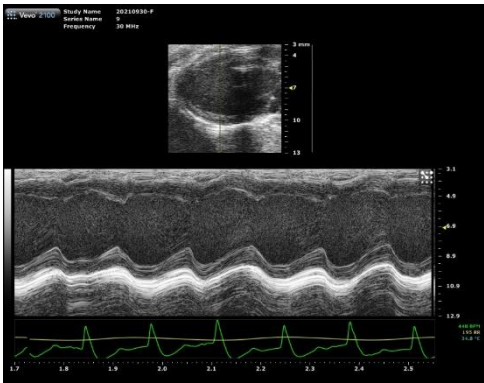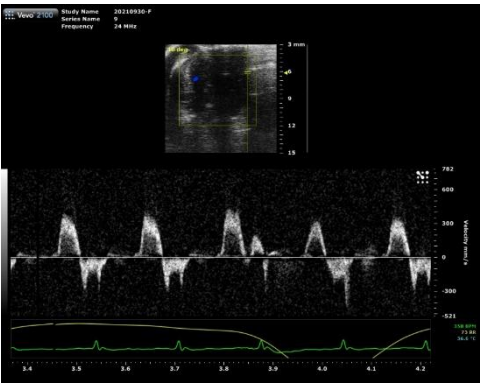

Supplement: Supplementary file 1 [file DataSheet2.PDF]
