# Supplementary material for: Hydrogen sulfide alleviates uremic cardiomyopathy by regulating PI3K/PKB/mTOR-mediated overactive autophagy in 5/6 nephrectomy mice
Source: Front Pharmacol. 2022 Dec 15;13:1027597. doi: 10.3389/fphar.2022.1027597 (PMC9797717; doi:10.3389/fphar.2022.1027597)
Supplement: Supplementary file 3 [file DataSheet4.PDF]

Representative Western blot images of LC3, becline-1, P62, P-PI3K/PI3K, P-PKB/PKB and P-mTOR/mTOR in the heart tissue of the 5 groups of mice. (Fig6)

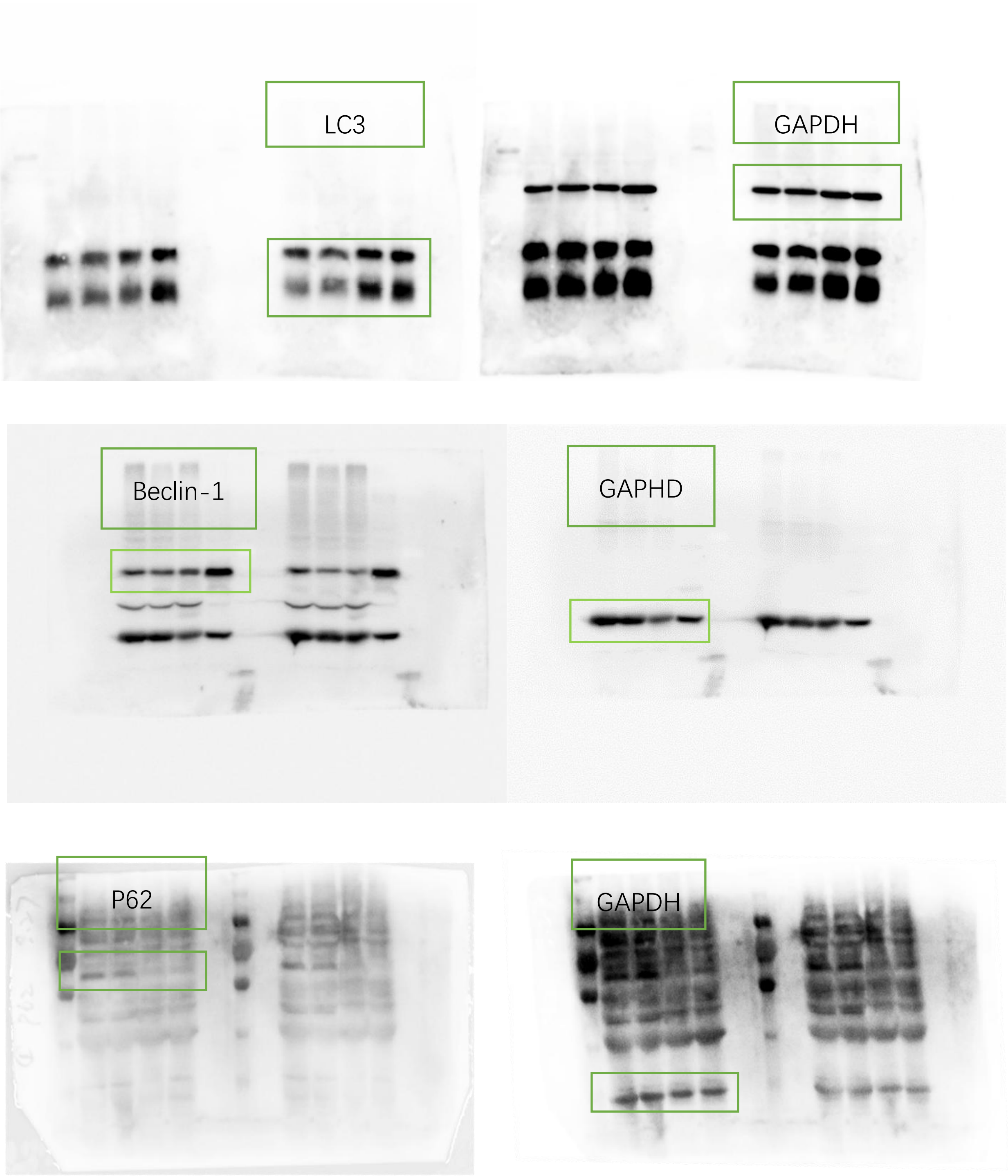

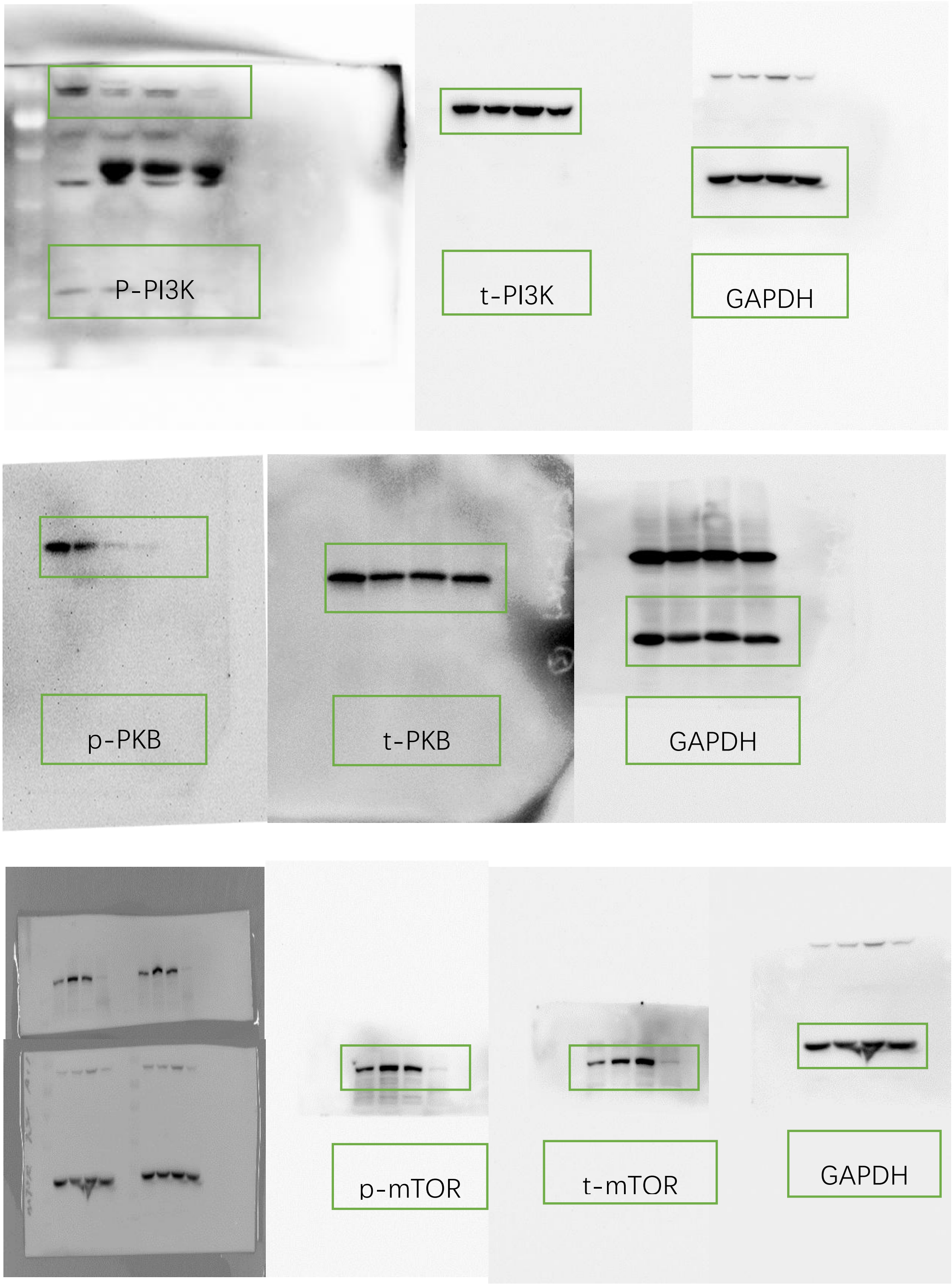
P-PI3K
t-PI3K
GAPDH
p-PKB
t-PKB
GAPDH
p-mTOR
t-mTOR
GAPDH
