# Supplementary material for: Hydrogen sulfide alleviates uremic cardiomyopathy by regulating PI3K/PKB/mTOR-mediated overactive autophagy in 5/6 nephrectomy mice
Source: Front Pharmacol. 2022 Dec 15;13:1027597. doi: 10.3389/fphar.2022.1027597 (PMC9797717; doi:10.3389/fphar.2022.1027597)
Supplement: Supplementary file 5 [file DataSheet1.PDF]

1. Representative Western blot images of CSE, LC3, becline-1, P62, P-PI3K/PI3K, P-PKB/PKB and P-mTOR/mTOR in the heart tissue of the 5 groups of mice. (Fig2-3)

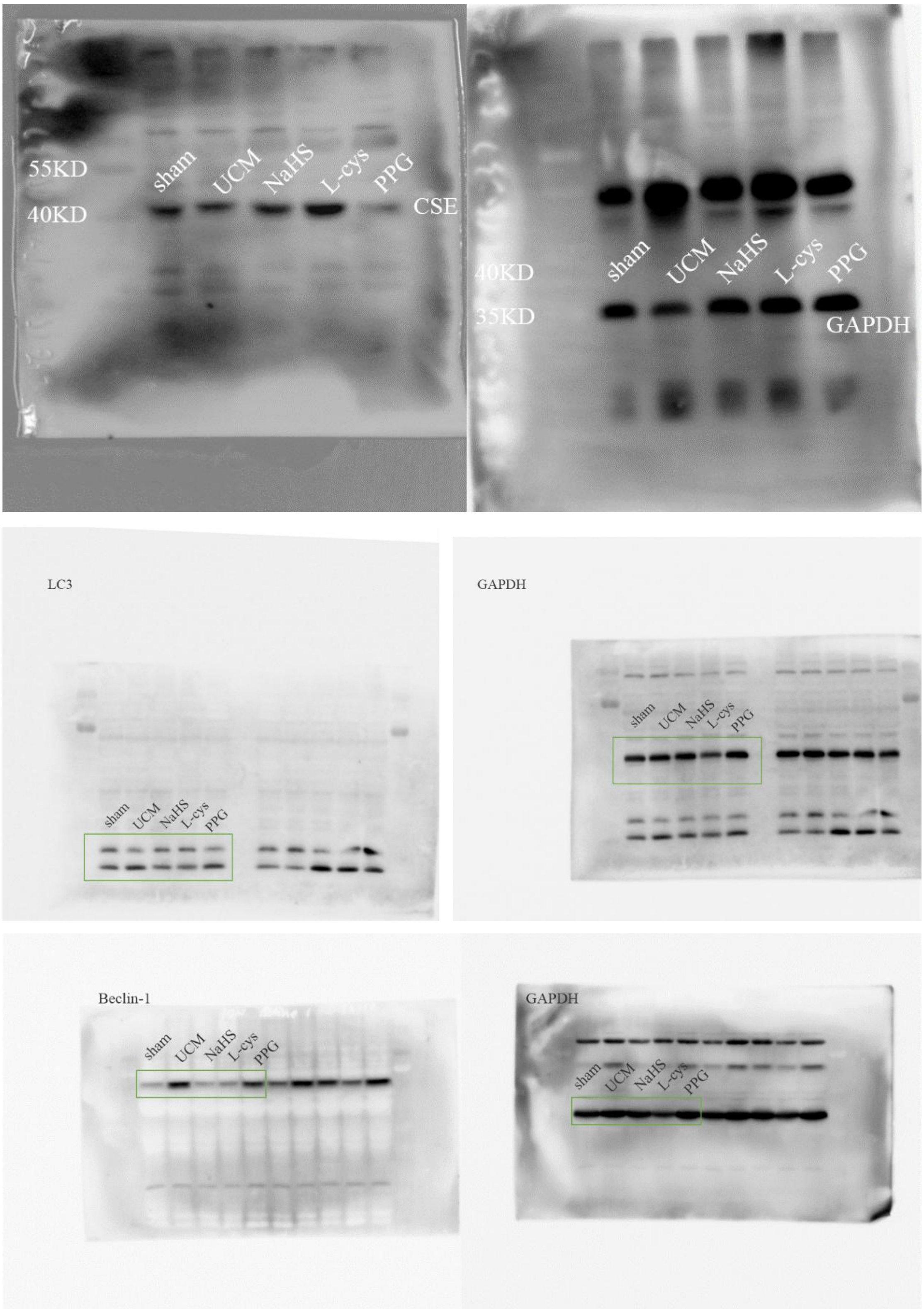

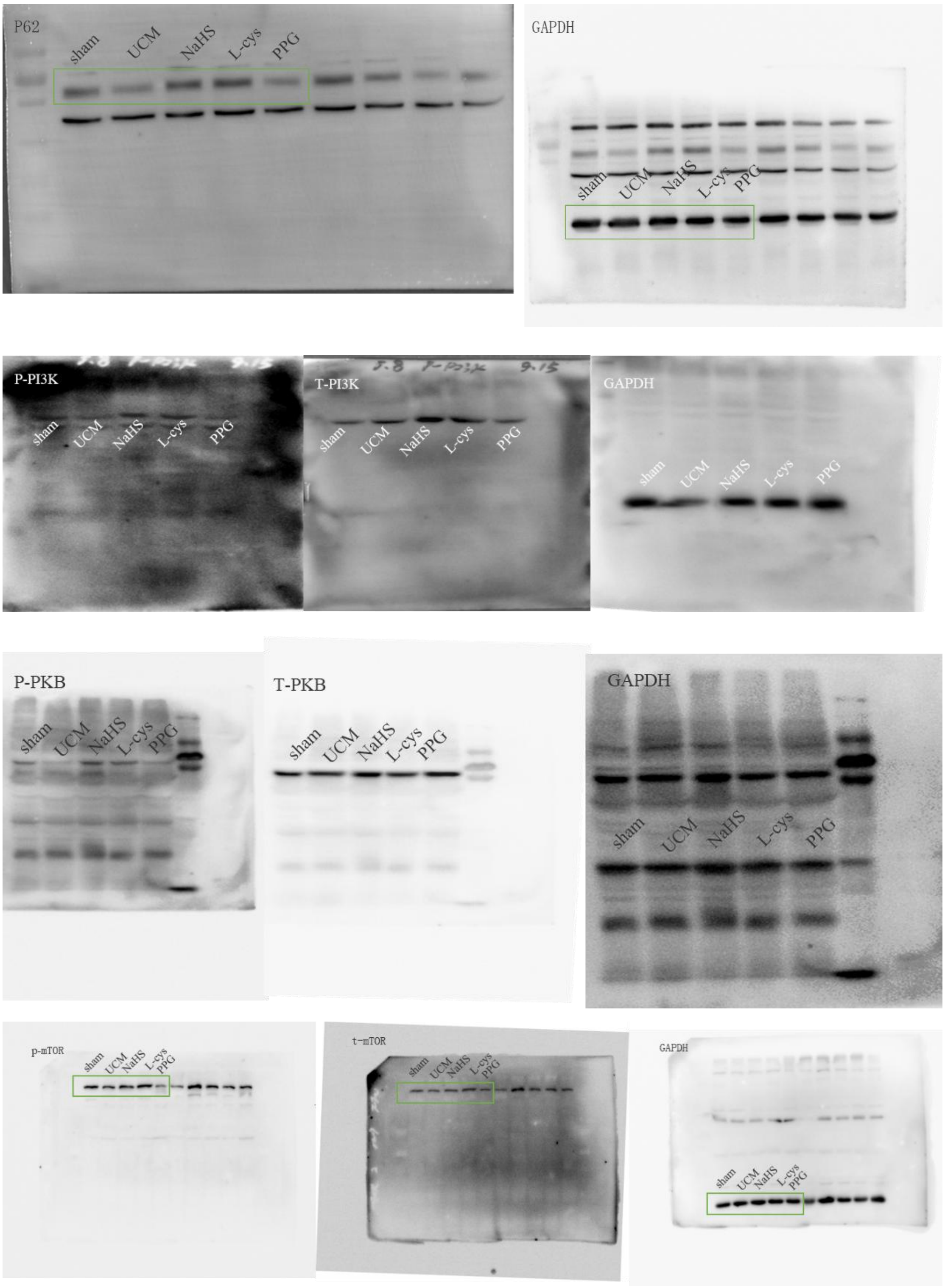
P62
sham UCM NaHS L-cys PPG
GAPDH
sham UCM NaHS L-cys PPG
P-PI3K
sham UCM NaHS L-cys PPG
T-PI3K
sham UCM NaHS L-cys PPG
GAPDH
sham UCM NaHS L-cys PPG
P-PKB
sham UCM NaHS L-cys PPG
T-PKB
sham UCM NaHS L-cys PPG
GAPDH
sham UCM NaHS L-cys PPG
p-mTOR
sham UCM NaHS L-cys PPG
t-mTOR
sham UCM NaHS L-cys PPG
GAPDH
sham UCM NaHS L-cys PPG

2. Representative Western blot images of Serum uremia on the expressions of CSE protein and autophagy-associated protein (LC3, P62, Beclin-1). (Fig5)

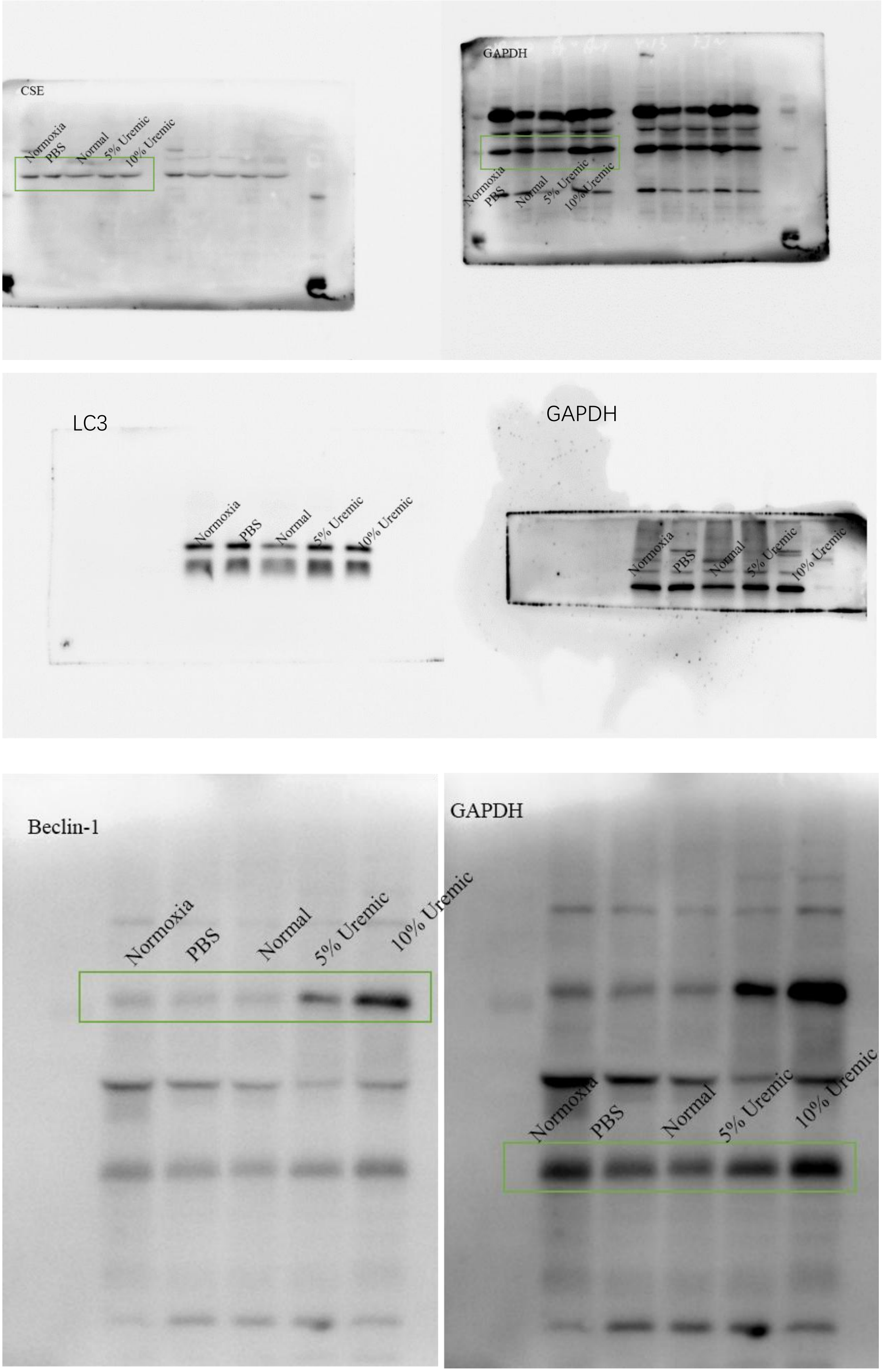

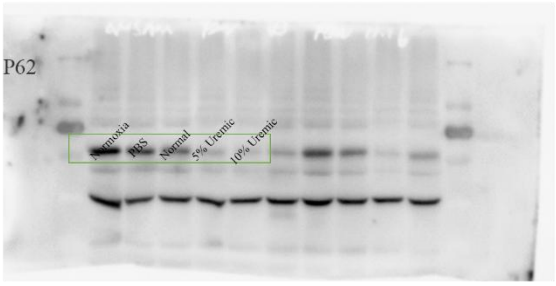
P62
Normoxia
PBS
Normal
5% Uremic
10% Uremic

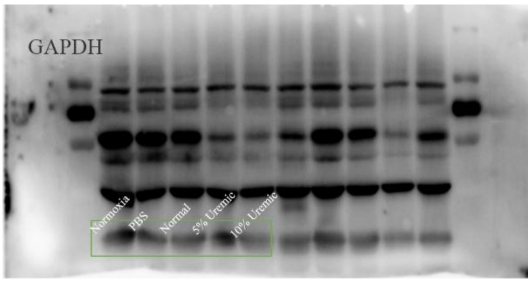
GAPDH
Normoxia
PBS
Normal
5% Uremic
10% Uremic

3. Representative Western blot images of CSE protein levels in the different concentrations of PPG in H9C2 cardiomyocytes. (Fig 6)

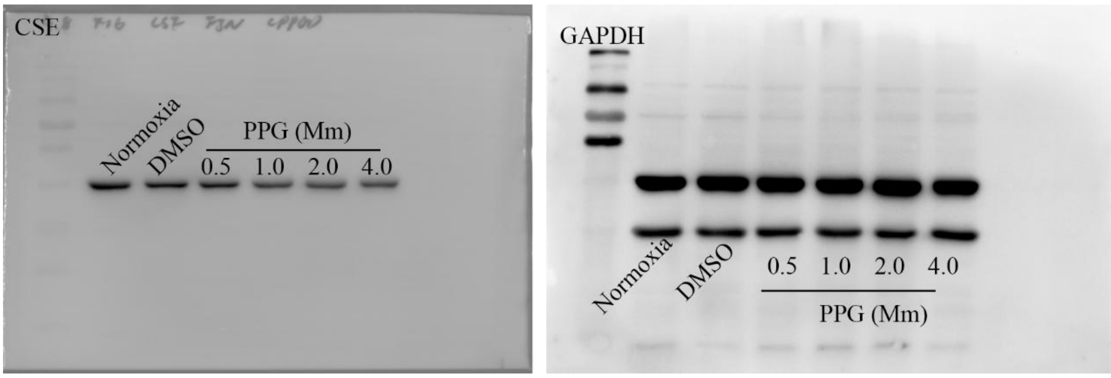

4. Representative Western blot images of LC3, becline-1, P62 P-PI3K/PI3K, P-PKB/PKB, and P-mTOR/mTOR in 4 groups of H9C2 cardiomyocytes (Normoxia, 10% Uremic, 10% Uremic+NaHS, 10% Uremic+PPG). (Fig 6)

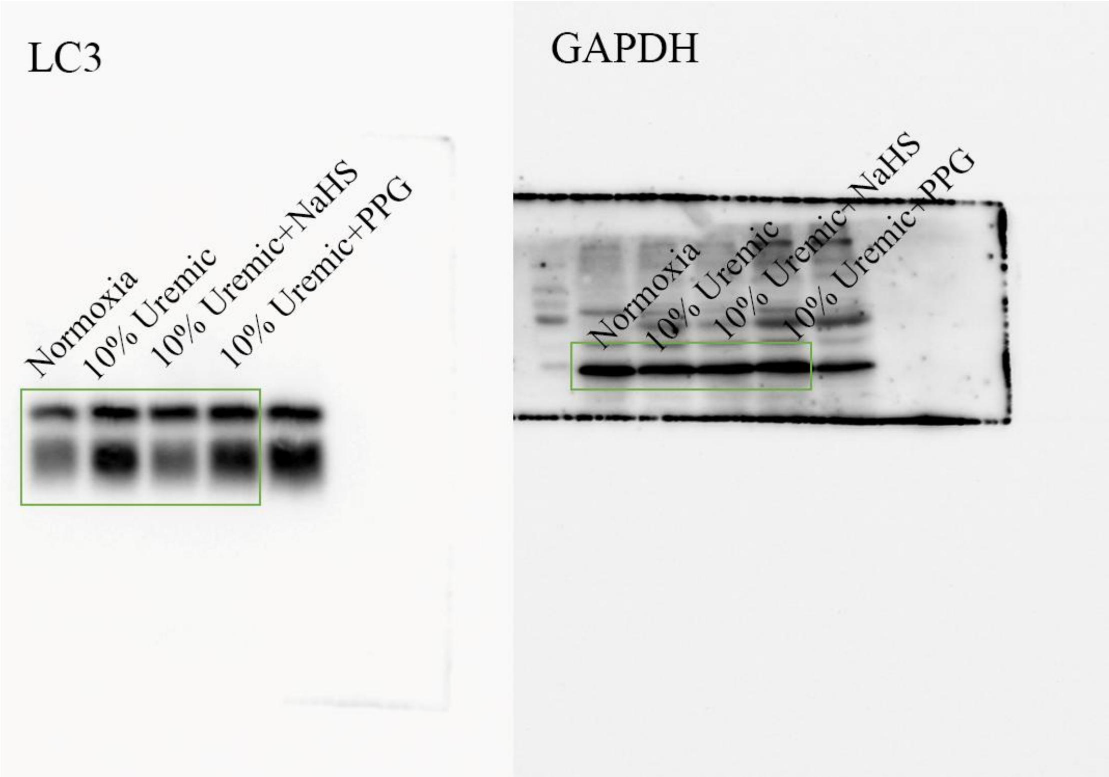

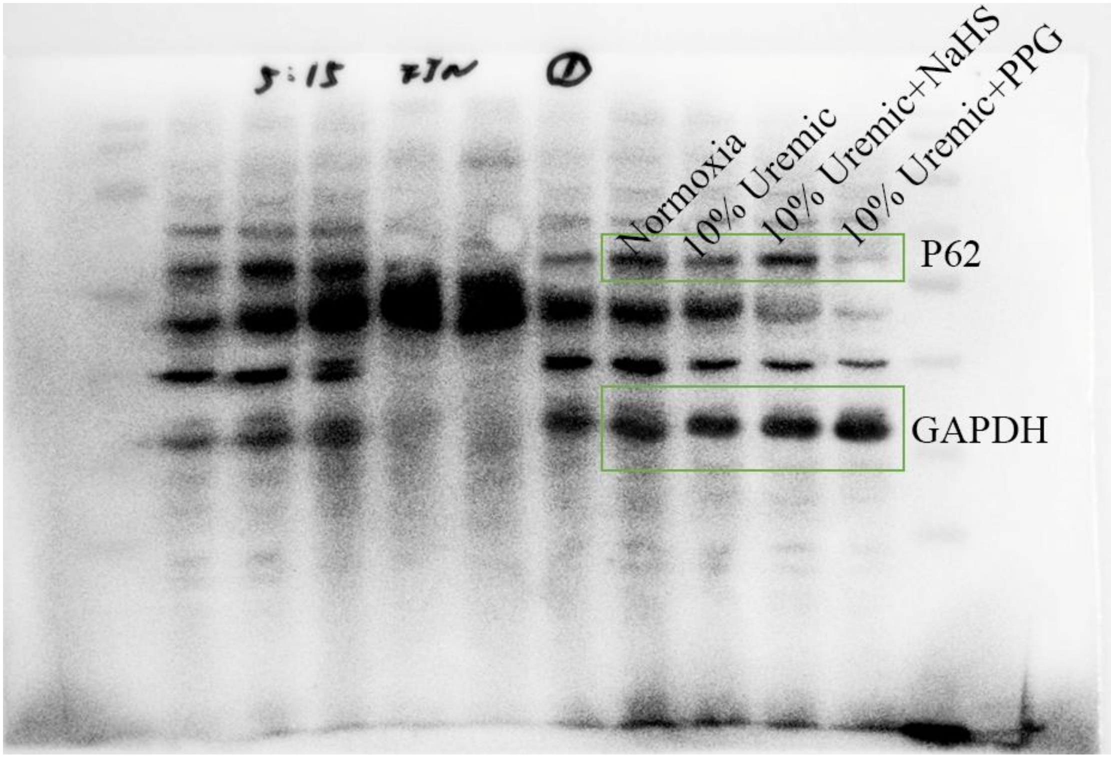
Normoxia
10% Uremic
10% Uremic+NaHS
10% Uremic+PPG
P62
GAPDH

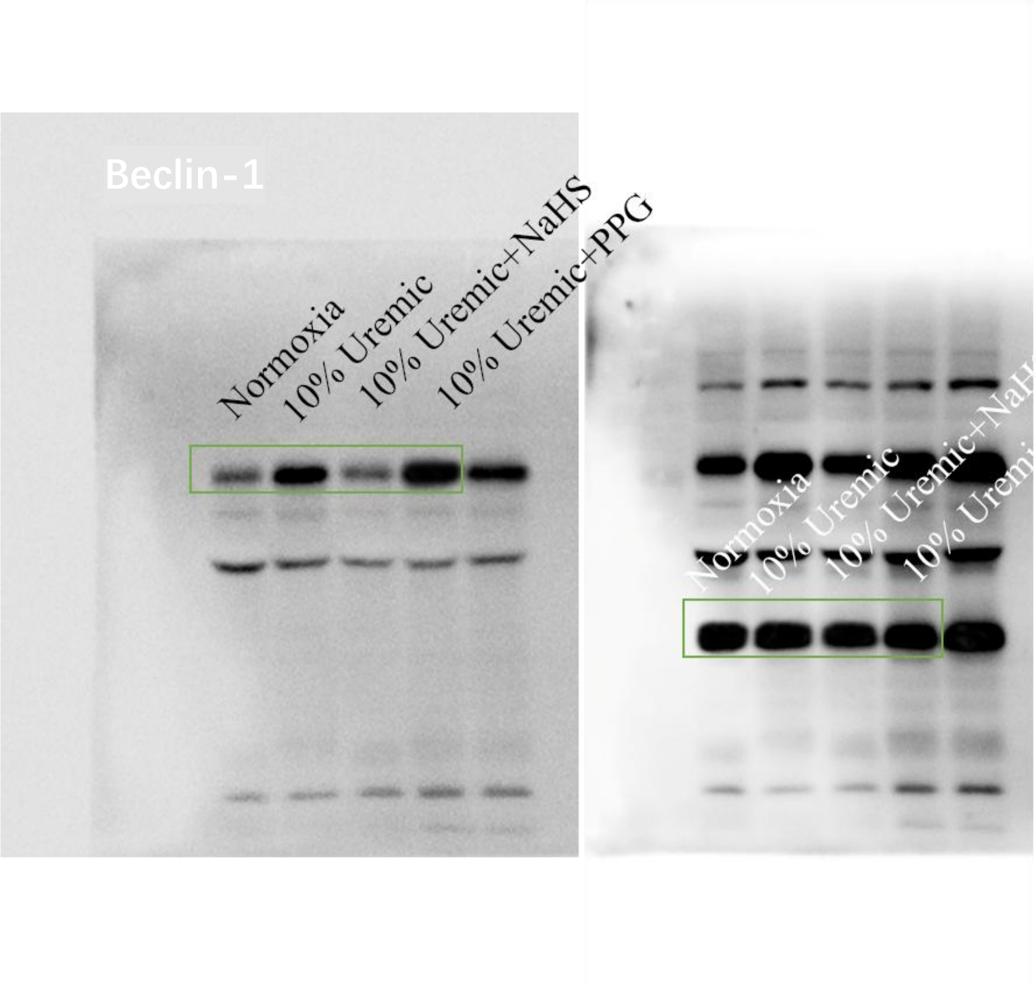
Beclin-1
Normoxia
10% Uremic
10% Uremic+NaHS
10% Uremic+PPG
Normoxia
10% Uremic
10% Uremic+NaHS

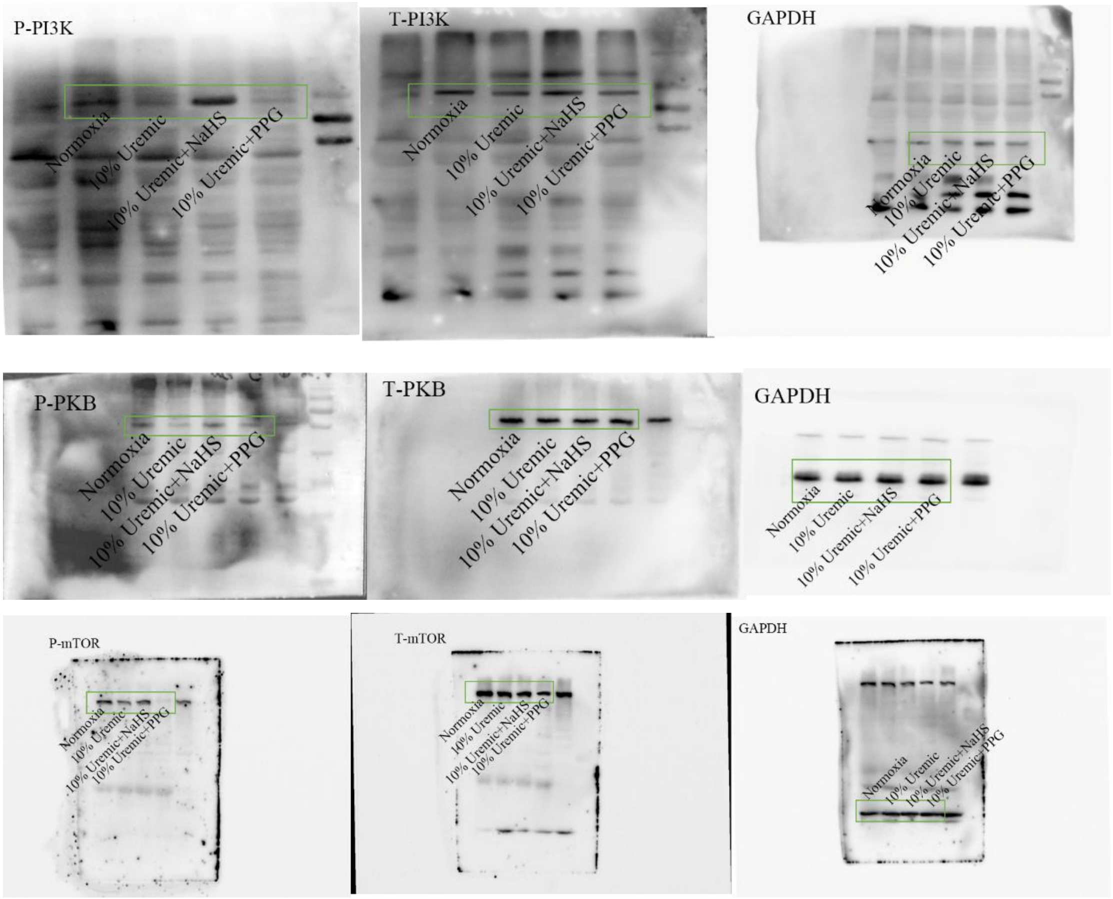
P-PI3K
T-PI3K
GAPDH
P-PKB
T-PKB
GAPDH
P-mTOR
T-mTOR
GAPDH
Normoxia
10% Uremic
10% Uremic+NaHS
10% Uremic+PPG

6. Representative Western blot images of LC3, becline-1, P62 P-PI3K/PI3K, P-PKB/PKB, and P-mTOR/mTOR in 4 groups of H9C2 cardiomyocytes (Normoxia, 10% Uremic, 10% Uremic+NaHS, 10% Uremic+ NaHS+Ly294002). (Fig 7).

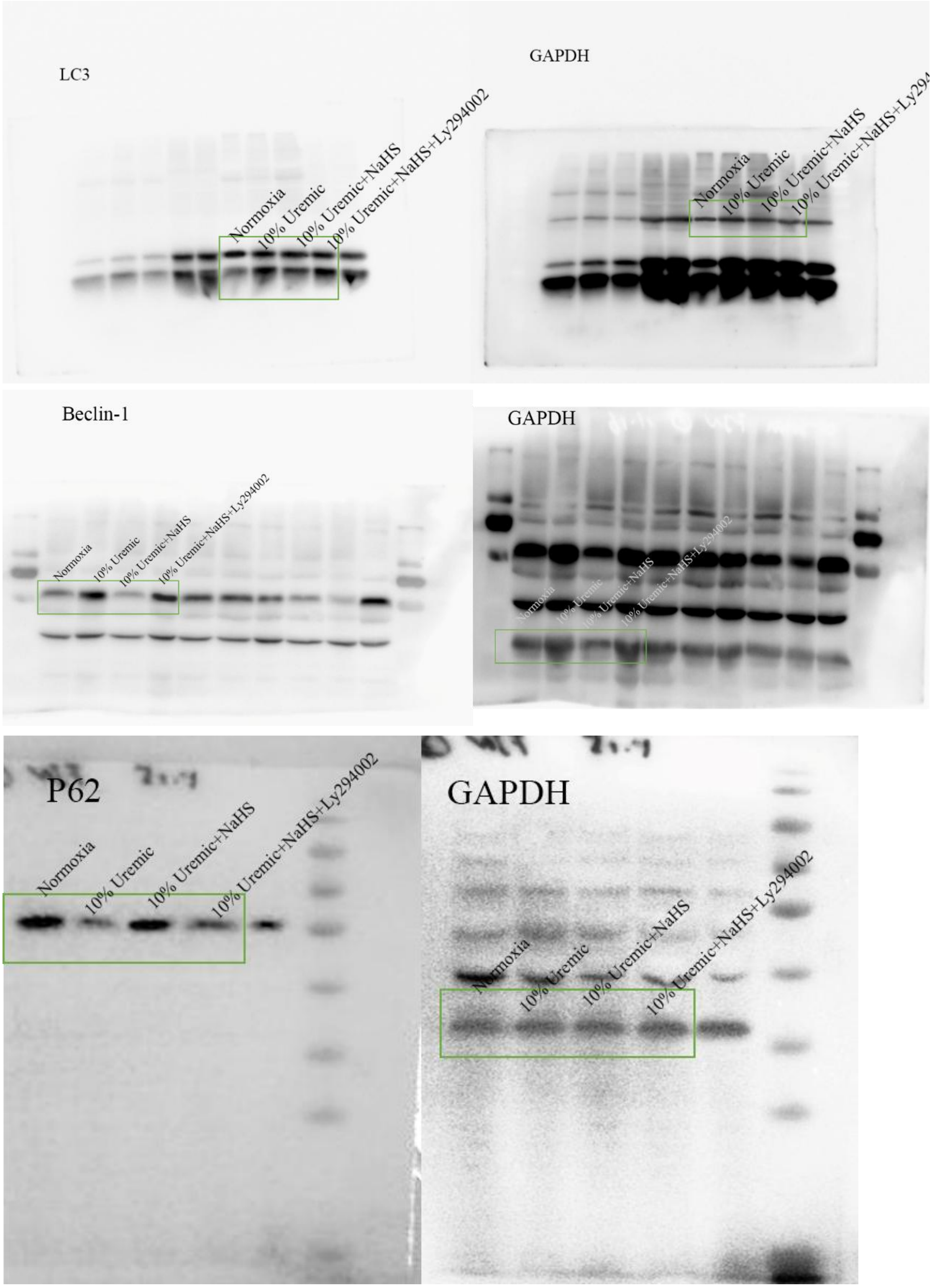

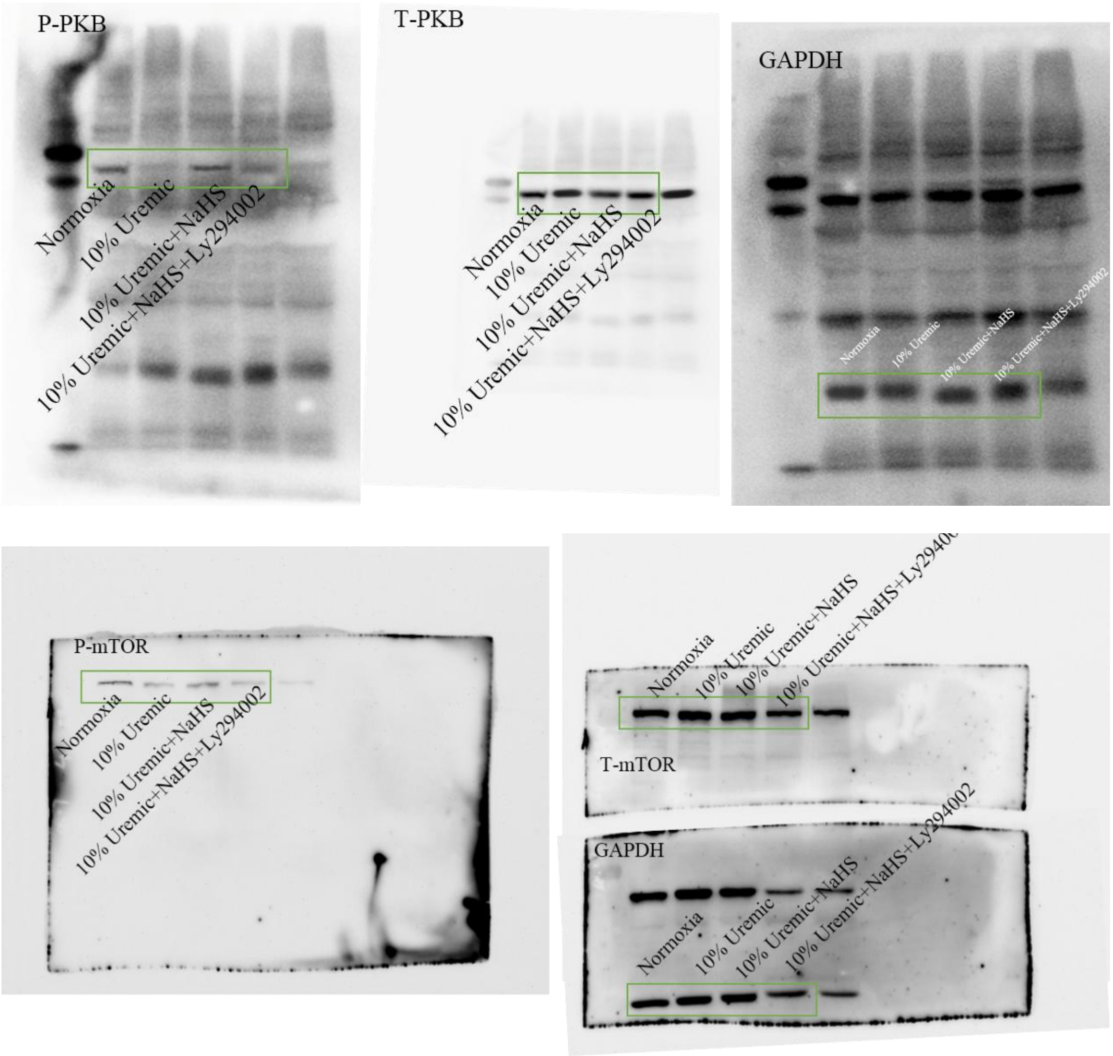
P-PKB
Normoxia
10% Uremic
10% Uremic+NaHS
10% Uremic+NaHS+Ly294002
T-PKB
Normoxia
10% Uremic
10% Uremic+NaHS
10% Uremic+NaHS+Ly294002
GAPDH
Normoxia
10% Uremic
10% Uremic+NaHS
10% Uremic+NaHS+Ly294002
P-mTOR
Normoxia
10% Uremic
10% Uremic+NaHS
10% Uremic+NaHS+Ly294002
Normoxia
10% Uremic
10% Uremic+NaHS
T-mTOR
GAPDH
Normoxia
10% Uremic
10% Uremic+NaHS
10% Uremic+NaHS+Ly294002
